# Accurate Localization of Linear Probe Electrode Arrays across Multiple Brains

**DOI:** 10.1523/ENEURO.0241-21.2021

**Published:** 2021-11-10

**Authors:** Liu D. Liu, Susu Chen, Han Hou, Steven J. West, Mayo Faulkner, The International Brain Laboratory, Michael N. Economo, Nuo Li, Karel Svoboda

**Affiliations:** 1 Janelia Research Campus, Howard Hughes Medical Institute, Ashburn, VA 20147; 2 Baylor College of Medicine, Houston, TX 77030; 3 University College London, London WC1E 6BT, United Kingdom; 4 Sainsbury Wellcome Centre, London W1T 4JG, United Kingdom; 5 Boston University, Boston, MA 02215

**Keywords:** electrode localization, lightsheet imaging, Neuropixels probes, serial blockface 2P imaging

## Abstract

Recently developed probes for extracellular electrophysiological recordings have large numbers of electrodes on long linear shanks. Linear electrode arrays, such as Neuropixels probes, have hundreds of recording electrodes distributed over linear shanks that span several millimeters. Because of the length of the probes, linear probe recordings in rodents usually cover multiple brain areas. Typical studies collate recordings across several recording sessions and animals. Neurons recorded in different sessions and animals thus have to be aligned to each other and to a standardized brain coordinate system. Here, we evaluate two typical workflows for localization of individual electrodes in standardized coordinates. These workflows rely on imaging brains with fluorescent probe tracks and warping 3D image stacks to standardized brain atlases. One workflow is based on tissue clearing and selective plane illumination microscopy (SPIM), whereas the other workflow is based on serial block-face two-photon (SBF2P) microscopy. In both cases electrophysiological features are then used to anchor particular electrodes along the reconstructed tracks to specific locations in the brain atlas and therefore to specific brain structures. We performed groundtruth experiments, in which motor cortex outputs are labeled with ChR2 and a fluorescence protein. Light-evoked electrical activity and fluorescence can be independently localized. Recordings from brain regions targeted by the motor cortex reveal better than 0.1-mm accuracy for electrode localization, independent of workflow used.

## Significance Statement

Recent advances in silicon electrode array recording technology dramatically increased probe length and the density of electrode sites. Specifically, Neuropixels probes span multiple regions of the mouse brain across 10-mm-long shanks. To localize recorded neurons, it is critical to localize recording sites. Here, we develop two workflows based on brain-wide imaging of probe tracks and analysis of electrophysiological landmarks in a standardized brain coordinate system. One workflow is based on lightsheet microscopy. The other workflow involves serial block-face two-photon (SBF2P) microscopy. We evaluate these workflows based on ground truth experiments. We show that electrodes, and thereby recorded neurons, can be localized to better than 100 μm.

## Introduction

Behavior is produced by organized multiregional neural circuits. A major goal of neuroscience is to understand behavior in the context of brain-wide maps of neural activity at the single cell level. Tracking how neural activity propagates across multiregional neural circuits requires measurements of neural activity on the scale of milliseconds and brain-wide reach, which is provided by extracellular recordings. Many nuclei of the rodent brain are smaller than 0.5 mm, such as parts of thalamic nuclei that communicate with defined regions of frontal cortex (Guo et al., 2018; Harris et al., 2019) and nuclei related to specific orofacial movements in the medulla (Moore et al., 2014; McElvain et al., 2018). This fine scale parcellation of the brain necessitates precise 3D localization of recorded neurons in a standardized brain coordinate system. Moreover, since current extracellular electrodes sample only a sparse subset of neurons and brain areas in one experiment, activity maps of the entire multiregional circuit have to be assembled across recordings from multiple experimental sessions and across multiple animals performing the same behavior. The somata of recorded neurons are near (<0.1 mm) recording electrodes (Henze et al., 2000), localizing neurons is therefore equivalent to localizing extracellular electrodes.

Classical systems neuroscience experiments have often combined neurophysiological measurements with anatomic and functional mapping to map recording locations. For example, studies in the mouse barrel cortex routinely focus on specific barrel columns that process information from one identified whisker (Yu et al., 2016; Petersen, 2019). Individual barrel columns are recognizable in histologic preparations as a ring-like arrangement of cell bodies (the barrel). Small electrolytic lesions can be used to mark the tissue near the electrode for localization in histologic material (Sofroniew et al., 2015). In addition, deflection of one whisker excites neurons mainly in the corresponding barrel column, which can be used to identify specific barrels during *in vivo* recordings (Yu et al., 2016; Petersen, 2019). Similar approaches are widely used in recordings from other brain regions that have been deeply explored using anatomic and/or physiological mapping techniques, such as the sensory thalamus and visual cortex (Hubel and Wiesel, 1959; Siegle et al., 2021). However, most of the mammalian brain has not been analyzed at comparable levels of detail. Many brain areas do not have finely mapped sensory or motor maps, nor do they contain clear cytoarchitectural features, such as barrels, that could be used for alignment of neurophysiological measurements across multiple brains. A more general method for localizing electrodes in a standardized coordinate system is required.

This need is especially acute for recently developed Neuropixels probes. The probes are commercially available and have been rapidly adopted by many laboratories (Evans et al., 2018; Krupic et al., 2018; Allen et al., 2019; Bennett et al., 2019; Gardner et al., 2019; Kostadinov et al., 2019; Musall et al., 2019; Steinmetz et al., 2019; Trautmann et al., 2019; Böhm and Lee, 2020; Luo et al., 2020; Sauerbrei et al., 2020; Siegle et al., 2021). Neuropixels probes (Jun et al., 2017; Steinmetz et al., 2021) provide recordings across 960 recording electrodes distributed over 9.6 mm shanks. Because of their long shanks, Neuropixels recordings naturally span multiple brain areas (Jun et al., 2017; Allen et al., 2019; Steinmetz et al., 2019; Siegle et al., 2021). Neuropixels probes lack the electronic elements to pass the large currents that are required to produce electrolytic lesions. In the workflows presented here, we focused on localization of Neuropixels probes in a standardized coordinate system, but the same methods can be applied to other linear probes.

In larger animals, it has been possible to localize electrodes in the intact brain using x-ray, MRI, or ultrasound imaging (Glimcher et al., 2001; Matsui et al., 2007; Cox et al., 2008). But these methods require specialized instruments and have limited resolution and contrast. These methods are also difficult to combine with acute recordings in head-restrained mice, where the probes are inserted and removed from the brain in each experimental session.

Localizing electrodes on linear probes has been achieved by labeling silicon probes with fluorescent dye and *post hoc* analysis of the recorded tissue using histologic methods (DiCarlo et al., 1996; Jensen and Berg, 2016; Guo et al., 2017; Salatino et al., 2017), aided by identifying known electrophysiological features of specific anatomic locations (Jun et al., 2017; Allen et al., 2019; Steinmetz et al., 2019; Siegle et al., 2021). Our methods are building on this approach and assess the accuracy of such workflows using groundtruth experiments.

In addition to localizing electrodes in individual experiments, recording locations need to be aligned across experiments and brains. The structure of each brain differs, even for isogenic animals (Kovacević et al., 2005), and brains deform in an inhomogeneous manner when extracted from the skull and when undergoing various histologic procedures. To aggregate recordings across different brains, recording locations have to be precisely localized in individual brains and warped to a standard brain coordinate system.

Here, we evaluate workflows based on two *post hoc* imaging methods for localizing electrodes in Neuropixels probe recordings. One workflow is based on brain clearing (Ueda et al., 2020a,b) and whole-brain imaging using widely available selective plane illumination microscopy (SPIM; also referred to as “lightsheet microscopy”; Huisken and Stainier, 2009; Power and Huisken, 2017). Turnkey SPIM microscopes are available at imaging cores of many universities. The second workflow, used by the International Brain Laboratory (The International Brain Laboratory, 2017; Wool and The International Brain Laboratory, 2020), is based on serial block-face two-photon (SBF2P) microscopy (Portera-Cailliau et al., 2005; Ragan et al., 2012; Economo et al., 2016), which relies on relatively expensive and customized instrumentation, but produces images that are easily aligned to a widely used standardized brain coordinate system (common coordinate framework; CCF v3; Lein et al., 2007; Oh et al., 2014; Wang et al., 2020).

In both workflows, probe tracks are first reconstructed in a standardized coordinate system, and then individual electrodes are localized along the track using electrophysiological features that identify brain structures in standard brain atlases. The workflows are distinguished by the histologic, imaging, and alignment procedures. We performed groundtruth measurements using optogenetic and fluorescent tagging of axonal pathways. These groundtruth measurements reveal the accuracy of electrode alignment to be better than 0.1 mm for both workflows.

## Materials and Methods

### Terminology

Allen Mouse CCF: standard mouse brain coordinate system.

Allen Anatomical Template (AAT): image stack based on background fluorescence corresponding to the CCF (http://download.alleninstitute.org/informatics-archive/current-release/mouse_ccf/average_template/).

Allen Reference Atlas (ARA): segmentation of the AAT into anatomic compartments (http://download.alleninstitute.org/informatics-archive/current-release/mouse_ccf/annotation/ccf_2017/).

Template MRI volume (MRI3D): MRI volume for male VGAT-ChR2-eYFP mice from the Mouse Imaging Center at The Hospital for Sick Children in Toronto (http://repo.mouseimaging.ca/repo/for_svoboda_hhmi/).

Probe: Neuropixels probe with 960 electrodes (384 recorded at the same time).

Electrode: one recording site on the Neuropixels probe.

### Surgeries and animals

All animal experiments adhered to the guidelines set by the Janelia Research Campus Institutional Animal Care and Use Committee. Nine VGAT-ChR2-eYFP (JAX 014548, >P60, all male; Zhao et al., 2011) and seven wild-type C57BL/6 mice (>P60, five male and two female) were used in this study. The details of the surgery procedure can be found elsewhere (Guo et al., 2014b; dx.doi.org/10.17504/protocols.io.bcrsiv6e). Briefly, mice underwent stereotaxic surgery to implant headbars for head-fixation for electrophysiological recordings. The skull was made clear for photostimulation experiments (Guo et al., 2014a). The skin and periosteum were removed, and a thin layer of cyanoacrylate (Krazy glue) was applied to attach the headbar and cover the exposed skull. A layer of clear dental acrylic (Lang Dental) was then applied on top of cyanoacrylate and formed a chamber around the skull to contain the ground wire and artificial CSF (aCSF) during electrophysiological recordings. The animals received at least 3 d of rest after surgery before commencing experiments. Before electrophysiological recordings, we prepared a 0.6-mm diameter craniotomy to access the intended brain regions with Neuropixels probes (dx.doi.org/10.17504/protocols.io.9a8h2hw).

For the groundtruth experiments (Figs. 8, 9), 100 nl of AAV2/5-CamKII-hChR2-EYFP-WPRE virus (4.6 × 10^12^ titer; UNC) was injected into ALM (2.5 mm anterior, 1.5 mm lateral, 0.8 mm from the dura surface) of C57BL/6J mice (*n* = 7; dx.doi.org/10.17504/protocols.io.bctxiwpn) in the same surgery with headbar implantation (Petreanu et al., 2009). Briefly, before the surgery, glass pipettes (Drummond Scientific Company) were pulled and sharpened to have a bevel of 35° and an opening of 20 μm at the tip. The sharpened pipette was filled from the back end with mineral oil and attached to the piston of a volumetric microinjection system (Narishige). The viral suspension was then suctioned through the tip of the pipette before injection. The skull over the injection site was thinned with a dental drill and punctured with the tip of the pipette. The pipette was inserted slowly (2 μm/s) to the desired depth. The virus was slowly (0.5 nl/s) injected to the desired location and the pipette was kept at the same location for 20 min after the end of the injection before retracted out of the brain. The virus was allowed to express for at least four weeks before electrophysiological recordings.

### Recordings

Electrophysiological recordings were made with Neuropixels probes (Neuropixels 1.0) in head-fixed mice performing an auditory delayed response task (Inagaki et al., 2018). Before insertion, the probe tip was painted with CM-DiI (dx.doi.org/10.17504/protocols.io.wxqffmw). Briefly, the Neuropixels probe was secured to a micromanipulator, and the back side of the probe was dipped into a 1 μl droplet of CM-DiI dissolved in ethanol (1 μg/μl). The ethanol was allowed to evaporate, and the CM-DiI was dried onto the back side of the tip. After painting with CM-DiI, the probe was attached to a micromanipulator (Sensapex) and inserted slowly (2–8 μm/s) into the brain through the craniotomy on the skull. Neuropixels probes have 960 electrodes, spanning 10 mm of tissue (Jun et al., 2017). The electrodes are grouped into three banks. The first bank contains the 384 electrodes that are closest to the tip, the second bank contains the next 384 electrodes, and the last bank contains the remaining 192 electrodes. Each probe has 384 recording channels that can be configured to record from the available electrodes. We used the most common configuration where the recordings were acquired from the first bank. The recordings span 3.84 mm from the probe tip. The mean insertion depth was 3.3 mm from the tip of the probe to the surface of the brain so that electrodes typically spanned the brain surface, which allows localization of the surface as an electrophysiological landmark in the workflow (Extended Data Fig. 7-2*A*). In recordings where we inserted >3.84 mm, we also acquired recordings (2 min) from the second bank of electrodes to localize the surface.

After reaching the desired recording depth, the probe was allowed to settle for 10 min before recording. Up to five probes were inserted during each recording session. The location of each penetration was recorded with respect to skull landmarks. Daily recording sessions lasted 1–2 h, and were repeated for up to 4 d in a craniotomy. At the end of each recording session, the probe was retracted and cleaned using Tergazyme and distilled water. The craniotomy was sealed with removable adhesive (Kwik-Cast, World Precision Instruments) and opened again before the next session of recording. Within a craniotomy, we ensured each insertion is separated by at least 250 μm at the point of insertion or the insertion angles differ by >10°. This procedure allowed clear separation of probe tracks from different sessions of recordings within a craniotomy (dx.doi.org/10.17504/protocols.io.8tphwmn).

For the groundtruth experiments we stimulated ChR2-expressing neurons with a 473-nm OBIS laser (Coherent Inc.) aimed at the center of the ALM (2.5 mm anterior, 1.5 mm lateral) through a single mode optic fiber. The peak power was 5 mW for a 2.5 mm (4σ) spot size. We stimulated with six 2-ms square pulses at 200-ms intervals, repeated every 5 s.

### Brain clearing

After the last recording session, mice were perfused transcardially with PBS and 4% paraformaldehyde (PFA). The brains were extracted from the skull and postfixed in 4% PFA at 4°C for 12 h before commencing the clearing procedure (dx.doi.org/10.17504/protocols.io.zndf5a6). Briefly, we used an alcohol-based delipidation procedure, where the brain was immersed in a 2-methyl-2-butanol (16% v/v) and 2-propanol (8% v/v) solution for 14 d. During delipidation, the brain was placed at 37°C with gentle shaking and a change of fresh solution daily. After the delipidation, the brain was washed with PBS for 1 d followed by refractive index (RI) matching in an iohexol-based solution (RI = 1.52) until it becomes visually transparent (Fig. 3*B*; Chi et al., 2018; Winnubst et al., 2019).

**Figure 1.**
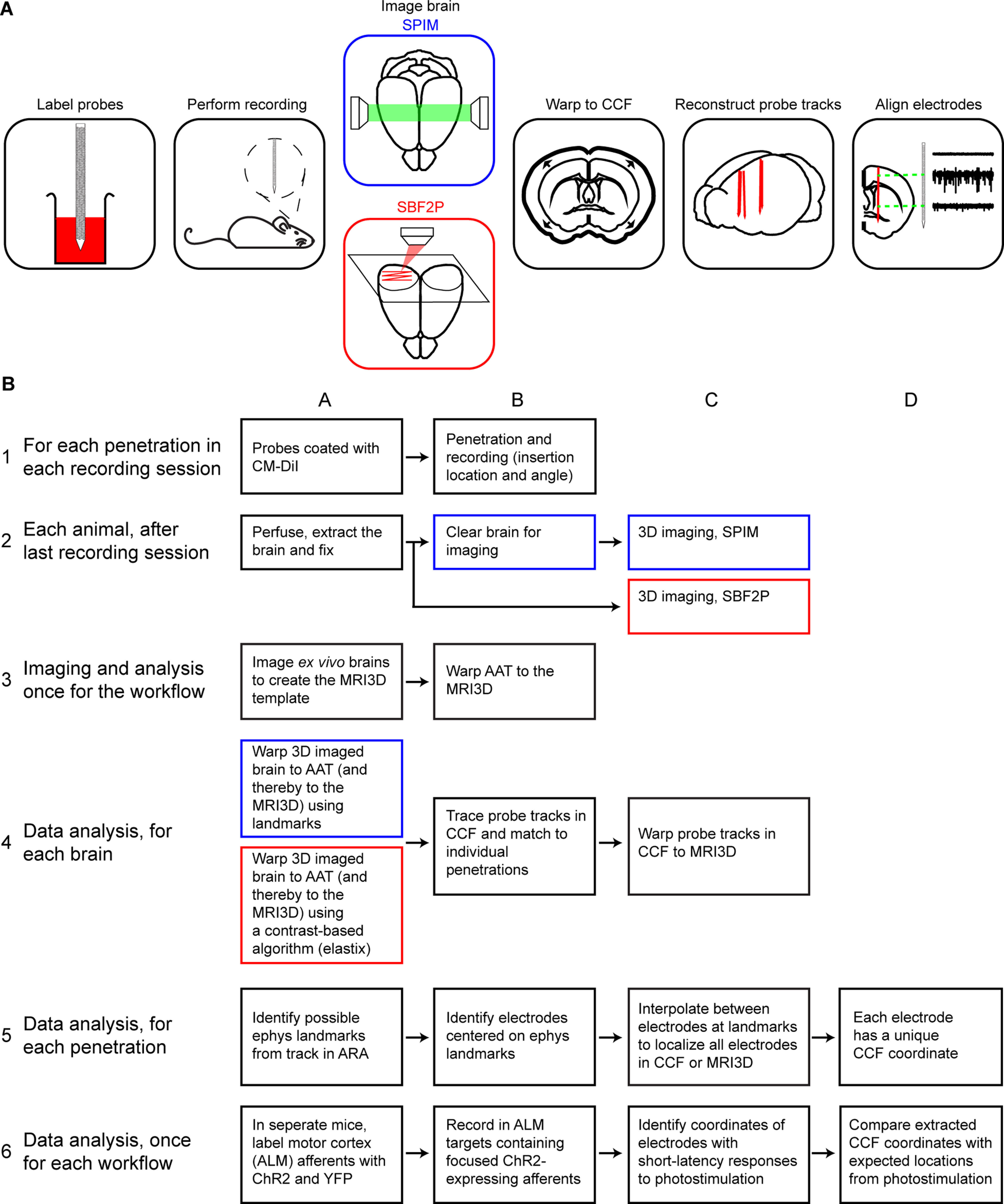
Electrode localization workflows. ***A***, Schematics of the workflows. Before each recording, probes are labeled with a fluorescent dye. After *in vivo* recordings, the brain is harvested. Fixed brains are processed and imaged in 3D. Blue box, whole-brain imaging with SPIM. Red box, Imaging with SBF2P. The imaged 3D volumes are warped to the CCF. The probe tracks are annotated in the 3D volume. Electrodes are localized along the track based on electrophysiological features that correspond to anatomic landmarks. ***B***, Detailed workflows for electrode localization. Blue boxes indicate steps specific to the SPIM workflow. Red boxes indicate steps specific to the SBF2P imaging workflow. Black boxes indicate steps that are shared by both workflows.

**Figure 2.**
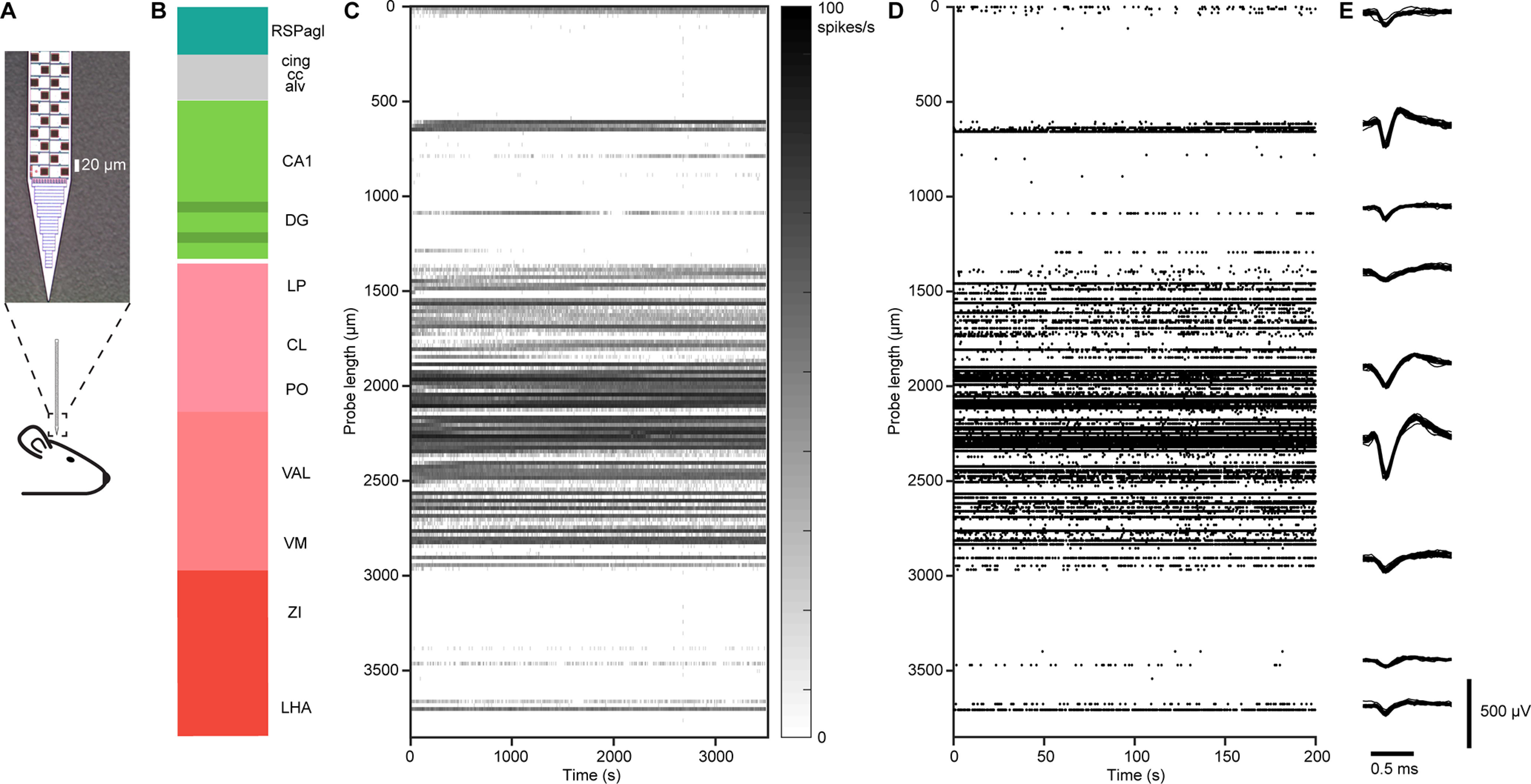
Neuropixels probe recording. ***A***, Schematic of the recording. Top, Image of the Neuropixels probe tip showing layout of electrodes. ***B***, An example penetration. The color along the probe track shows ARA compartments. ***C***, Spike rate showing multiunit activity (threshold −70 μV) across electrodes along the probe in ***B***, where 0 is the position of the most superficial electrode. The spike rate is binned at 10 s and 20 μm. The *y*-axis indicates the position of the spikes on the probe. Data from 3500 s of continuous recording. ***D***, First 200 s of spike rasters showing multiunit activity across electrodes along the probe in ***C***. Each event corresponds to a dot on the raster plot. Waveforms from nine example single units. A total of 25 overlaid waveforms each. The vertical position indicates an approximate position along the probe.

**Figure 3.**
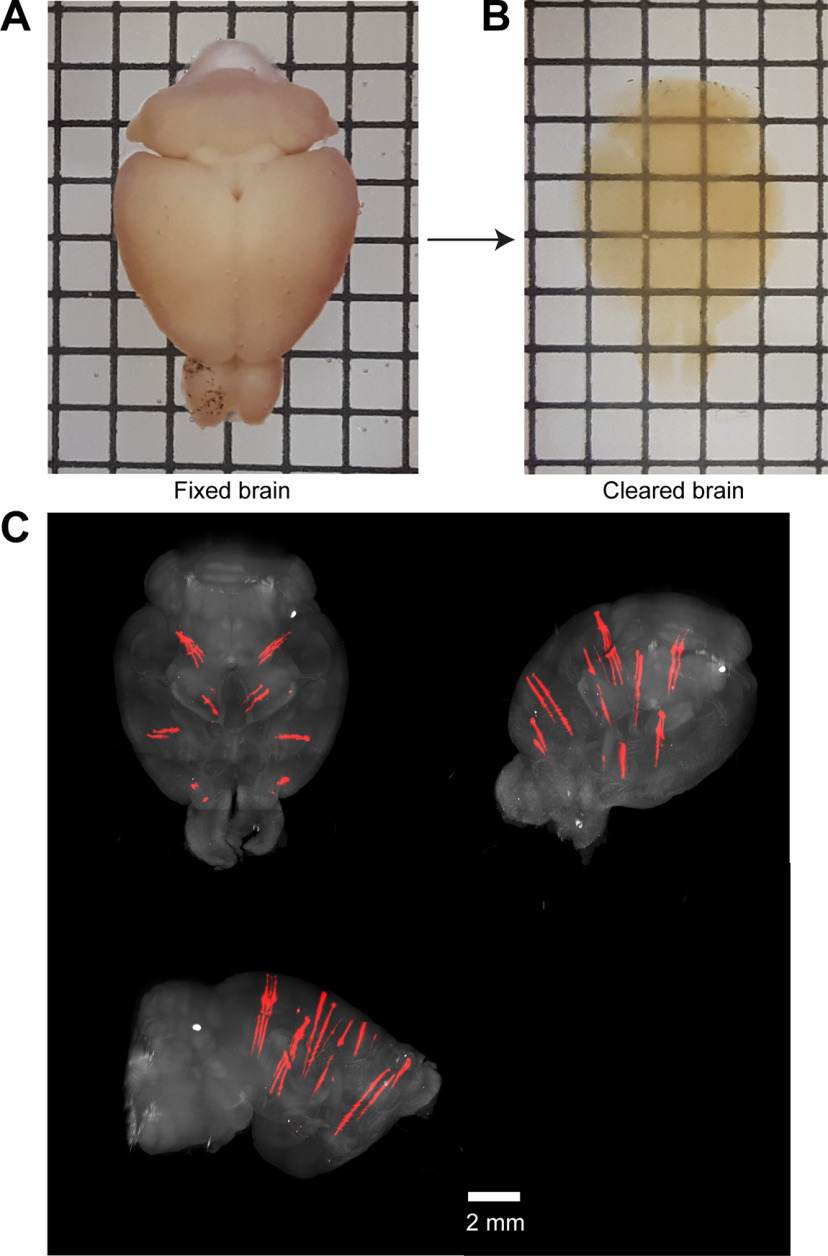
Brain clearing and probe tracks. ***A***, A fixed brain. The spacing between lines in the grid is 2.5 mm. ***B***, Cleared brain in RI matched solution. ***C***, Example 3D image volume acquired with SPIM. Red shows fluorescence from the CM-DiI labeled probe tracks. Image was taken from angles to show separated probe tracks. Example 3D image volume acquired with SPIM is available as Movie 1.

### Imaging

A variety of imaging methods have been used for whole-brain imaging. Classically the brain is cut into thin (e.g., 50 μm) sections which are then imaged using standard microscopy or slide scanners. Handling of large numbers of sections is labor intensive and error prone. In addition, sections are distorted, complicating assembly of 2D images into precisely aligned 3D volumes that can be registered to other 3D volumes.

These problems can be avoided with whole-brain clearing (Mano et al., 2018) and imaging without physical sectioning. We chose a tissue clearing method that results in mechanically robust specimens that are sufficiently transparent for whole-brain SPIM (Fig. 3*B*; Huisken and Stainier, 2009; Power and Huisken, 2017). The method preserves the fluorescence of fluorescent proteins and is compatible with immunohistochemistry (Winnubst et al., 2019). The cleared brain was imaged using a SPIM (Zeiss Lightsheet Z.1). We used 5× (NA 0.1) illumination objectives and a 5× (NA 0.16) detection objective. The brain was illuminated at 488 nm (50 mW). Each horizontal section was imaged with a 150-ms exposure. Images were acquired on two imaging channels. The 504- to 545-nm channel captured the eYFP and autofluorescence; a channel with a 585-nm longpass filter captured CM-DiI fluorescence. Both channels were also filtered with a notch filter for the 488 nm laser emission. The 3D image of the brain (v3D) was acquired by tiling image stacks in the horizontal plane. Each image stack was 2342 × 2342 μm in the horizontal (*XY*) plane and spanned the full brain in the dorsal-ventral axis (*Z*). A typical brain required 20–30 stacks, with 6–12% overlap between sections in *XY*. The spacing between each plane within a stack was 8 μm. The size of each v3D voxel was 1.22 × 1.22 × 8 μm (AP × ML × DV).

After imaging the stacks were stitched using image correlation in the overlap regions (Imaris Stitcher, Bitplane). Each horizontal section was downsampled by 5× to create the v3D used for warping to the Allen Anatomical Template (AAT), with voxel size 6.1 × 6.1 × 8 μm. The v3D volumes show the electrode tracks and distinct cytoarchitecture that can be used for alignment (Fig. 3*C*; Movie 1).

Movie 1. Example 3D brain volume imaged with SPIM showing the electrode tracks and distinct cytoarchitecture that can be used for alignment. 10.1523/ENEURO.0241-21.2021.video.1

Another whole-brain imaging system that avoids handling brain sections is based on SBF2P imaging. These microscopes image the tissue in a blockface configuration before cutting, thereby producing high-quality 3D image volumes (Ragan et al., 2012; Oh et al., 2014; Economo et al., 2016). Indeed, brain volumes imaged in this manner are the basis of the CCF. In three brains (a total of 14 penetrations), we performed the electrode localization procedure using SBF2P. Fixed brains were embedded in agar and imaged using a custom microscope at the Sainsbury Wellcome Centre (Ragan et al., 2012). The brains were coronally sectioned to 50 μm using a built-in custom-fitted Leica T1000 vibratome mechanism. The tissue was imaged with a 16× (NA 0.8) objective (Nikon). Tissue was excited with a Chameleon Ultra I two-photon laser at 920 nm (110 mW). Emission fluorescence was filtered with a 450/70-nm BrightLine single-band bandpass filter to capture the background fluorescence at 425–495 nm, Chroma ET525/50m filter to capture the eYFP fluorescence at 500–550 nm, and ET570 longpass filter to capture CM-DiI fluorescence at 570+ nm. The data were acquired on three channels with three multialkali Photomultiplier tubes at 750 V (Hamamatsu R10699). For each vibratome sectioned slice, imaging was done at two depths spaced 25 μm apart, the size of each resulting voxel was 4.4 × 4.4 × 25 μm (ML × DV × AP).

### 3D templates

For the images acquired with SPIM, we aligned the v3D to two template brains. First, the AAT (http://download.alleninstitute.org/informatics-archive/current-release/mouse_ccf/average_template/) is a high-resolution and high-contrast volume of SBF2P images, averaged over thousands of brains, where the contrast is based on autofluorescence. The AAT corresponds to a standardized brain coordinate system, the CCF v3 (Lein et al., 2007; Oh et al., 2014; Wang et al., 2020), and an atlas of anatomic structures, the Allen Reference Atlas (ARA; http://download.alleninstitute.org/informatics-archive/current-release/mouse_ccf/annotation/ccf_2017/; Dong, 2008; Wang et al., 2020). Second, we constructed a template MRI image volume (“MRI3D”) that is close to the unperturbed *in vivo* shape of the brain for the mice used in this study. Distance along the electrode better corresponds to distance in the MRI3D compared with the distorted AAT. The MRI3D therefore provides for more accurate placements of electrode sites in the absence of landmarks.

**Figure 4.**
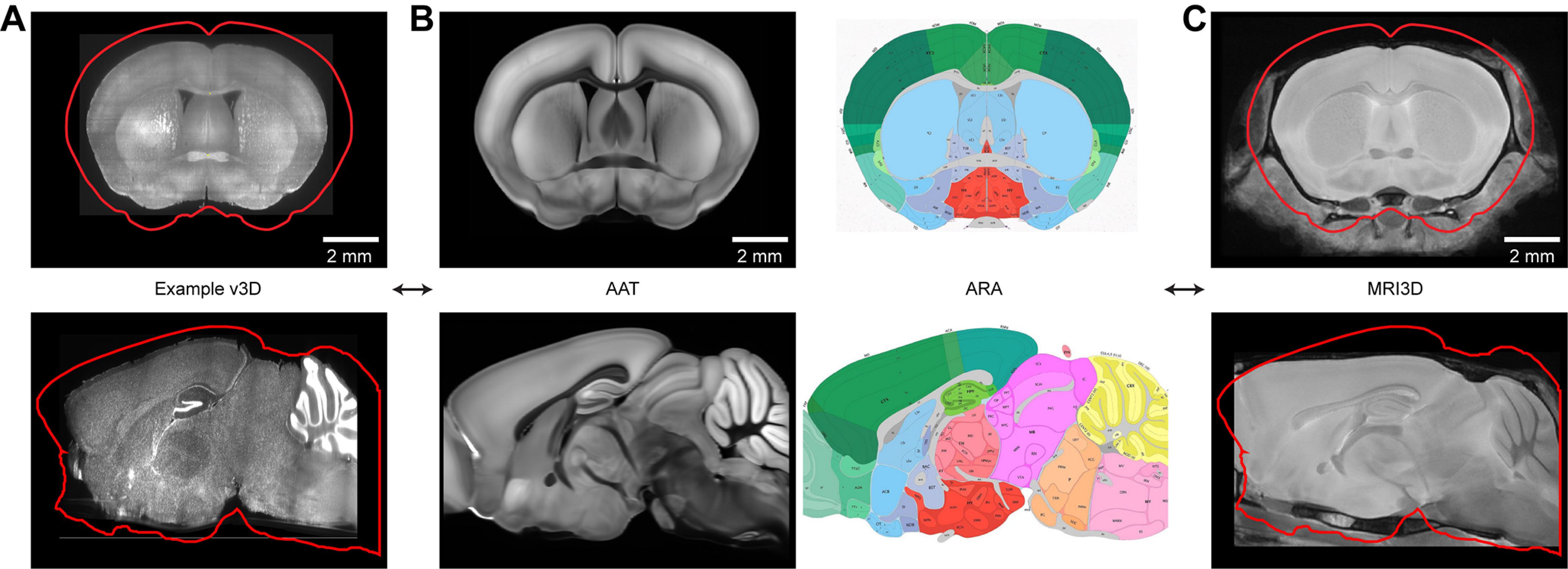
Brain volumes. ***A***, A coronal slice of an example v3D image volume. The red outline corresponds to the AAT. Bottom, Sagittal slice. ***B***, Same as ***A***, for the AAT and ARA. ***C***, Same as ***A***, for the template MRI3D image volume. Extended Data Figure 4-1 shows the example anatomic landmarks. Extended Data Figure 4-2 shows the variation in the MRI volumes across mice. Extended Data Figure 4-3 shows example warp fields.

The appearance of the AAT differs qualitatively from the v3D acquired with SPIM, because of differences in tissue preparation and imaging methods (compare Fig. 4*A*,*B*). In the SPIM workflow, we used a semi-manual landmark-based method to align brain volumes (BigWarp in ImageJ; Bogovic et al., 2016). Point correspondences between the v3D and AAT were manually determined. To transform the v3D into the AAT space, we used three dimensional thin plate spline interpolation (Duchon, 1977). We first determined the v3D↔CCF transformation by identifying a set of seven landmarks in both the v3D and AAT: the anterior and posterior ends of the corpus callosum (CC) along the midline; the meeting point of the anterior commissure along the midline, the genu of the facial cranial nerves to the brainstem in each hemisphere, and indentations from the medial cerebral arteries on the surface of each hemisphere (Table 1; Extended Data Fig. 4-1*A*). We warped individual v3Ds based on this initial set of landmarks, and then additional landmarks were placed as needed based on visual inspection (Extended Data Fig. 4-1*B*). The warping was performed iteratively after each landmark placement. A 3D volume typically requires 200–300 landmarks to define an accurate transformation (Extended Data Fig. 4-1*C*). A higher density of landmarks was placed around the brain locations containing probe tracks.

**Table 1:** Table of anatomic landmarks in the CCF.

Landmarks (Extended Data Fig. 4-1*A*)	CCF X (μm)	CCF Y (μm)	CCF Z (μm)
Anterior end of the CC along the midline	5700	3820	4240
Posterior end of the CC along the midline	5700	1780	7600
The meeting point of the anterior commissure along the midline	5700	5260	5160
Indentations for the medial cerebral arteries meeting the hippocampus in DV	600; 10,800	4500	7720
Genu of the facial cranial nerves to the brainstem	5100; 6300	5100	10820

10.1523/ENEURO.0241-21.2021.f4-1 Extended Data Figure 4-1 Example anatomical landmarks. ***A***, Landmarks for alignment (Table 1). The same landmarks (yellow) are identified in the AAT and v3D image volumes. ***B***, After placement of initial landmarks, warping was applied. Additional landmarks (yellow spheres) were then placed to better align the v3D and AAT. Higher densities of landmarks were placed near the probe tracks. ***C***, All landmarks of an example v3D and the AAT (black dots). Download Figure 4-1, TIF file.

10.1523/ENEURO.0241-21.2021.f4-2 Extended Data Figure 4-2 The variation in the MRI volumes is small across mice. SD as a percentage of the mean thickness measured in the nine VGAT-ChR2-eYFP mice used to generate the MRI3D template (Lerch et al., 2008). Download Figure 4-2, TIF file.

10.1523/ENEURO.0241-21.2021.f4-3 Extended Data Figure 4-3 Example warp fields for one coronal section and one sagittal section. Left, Averaged displacements to warp v3D image volumes onto the AAT. ML, displacements of the v3D along medial-lateral axis; DV, dorsal-ventral; AP, anterior-posterior. For example, in the top left image (ML), the v3D coronal section image has to be stretched laterally to align with the AAT (see Fig. 4*A*). The black lines on the sagittal sections at the bottom indicate the AP position of the coronal sections. Middle, SD of the displacements required to warp v3D image volumes onto the AAT (9 mice). Right, Displacements to warp the average MRI3D volume onto the AAT. Download Figure 4-3, TIF file.

The shapes of the 3D image volumes differ across individual mice and differ substantially from the AAT (Fig. 4*A*,*B*). After multiple recording sessions and penetrations, typically 16 per brain, damage at the insertion sites can cause local deformations. In addition, once extracted from the skull and cleared, fixed brains further deform in a nonuniform manner. For these reasons a relatively high number of landmarks is required.

The AAT was imaged *ex vivo* and is distorted compared with the brain *in vivo*. To warp the v3D into a shape resembling *in vivo* conditions, we imaged VGAT-ChR2-EYFP mice after fixation, but in the skull, using high-resolution MRI (Kovacević et al., 2005; Spencer Noakes et al., 2017). These image volumes are consistent across individual mice (Extended Data Fig. 4-2) and are less distorted compared with brains after extraction from the skull (Lerch et al., 2008; Ma et al., 2008; de Guzman et al., 2016). Individual MRI brains were averaged to obtain the template MRI image volume (“MRI3D”; Friedel et al., 2014; Nieman et al., 2018).

Why did we obtain our own MRI3D rather than use existing MRI mouse brain atlases? Depending on strain, sex, and age, mice show considerable variation in brain size and cortical thickness (∼15%), as reflected in existing mouse MRI atlases (Chen et al., 2006; Dorr et al., 2008; Johnson et al., 2010; Steadman et al., 2014; Scholz et al., 2016; Qiu et al., 2018). For assignment of electrodes over a 1-mm span, 15% distortions in the brain space would produce up to 0.15-mm errors in electrode placement.

A comparison of the MRI3D with AAT revealed that the AAT is enlarged compared with our *in vivo* conditions (Fig. 4) and distorted in a nonuniform manner (Extended Data Fig. 4-3). We established the AAT↔MRI3D mapping using the landmark-based method described above. Thus, any v3D warped into CCF also is automatically aligned to the MRI3D. Because the MRI3D approximately maintains the shape and size of the brain in the *in vivo* recording condition, it permits more accurate placement of the electrode sites (below).

**Figure 5.**
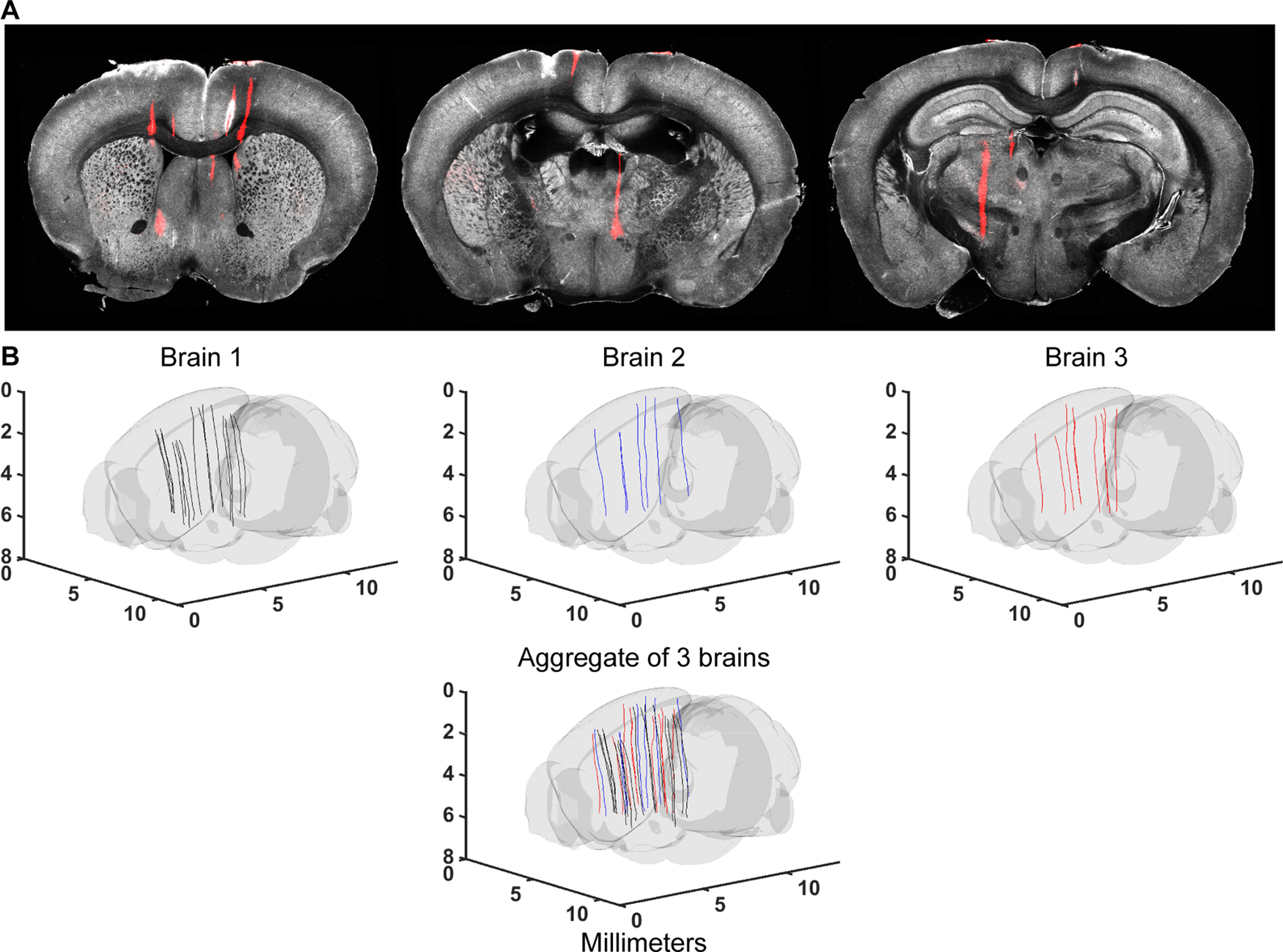
Brain volumes and probe tracks for the SBF2P workflow. ***A***, Example coronal sections from the SBF2P imaging. ***B***, Annotated probe tracks in the CCF for the three brains and an aggregate of probe tracks in the CCF. Different colors indicate tracks from different brains.

For mice imaged with SBF2P, the image contrast is similar to the AAT (Fig. 5*A*). Instead of using manually placed landmarks, the brain volumes were automatically aligned to the AAT using a contrast-based algorithm (Klein et al., 2010). The warping algorithm is based on an affine transform followed by a B-spline transform of the volume acquired on the background fluorescence channel to the AAT (Klein et al., 2010). We used similar Elastix parameters as in previous registrations to the AAT (Ragan et al., 2012) with the Advanced Mattes mutual information as the optimization metric between the v3D volume and the AAT (Mattes et al., 2003; Ragan et al., 2012). The registration was defined as an optimization of the affine and B-spline parameters that minimizes the discrepancy in contrast between the images. The registration was at multiple resolutions with the affine parameters optimized at four resolutions, and the B-spline parameters optimized at six resolutions (Lester and Arridge, 1999). The optimization was done in an iterative manner using the adaptive stochastic gradient descent optimizer at a maximum of 500 iterations at each resolution. (https://github.com/SuperElastix/elastix).

### Template MRI brain

The MRI imaging was performed in a high resolution 7T MRI at the Mouse Imaging Center at The Hospital for Sick Children in Toronto (Spencer Noakes et al., 2017). The animals were very slowly (1 ml/min) perfused with 4% PFA and MRI contrast enhancement agent Prohance (Gadoteridol, Bracco Diagnostics). After perfusion, the head was detached from the body and the skin removed from the skull. After 12 h of fixation with the brain inside the skull, the brains were kept in 1× PBS and 2 mm ProHance until ready to be imaged. The brains were imaged in the skull, where distortion from fixation is minimized (de Guzman et al., 2016). The resolution of the 3D stack is 40 × 40 × 40 μm.

Nine VGAT-ChR2-eYFP (JAX 014548, >P60, all male) mice contributed to the average image stack. The images of individual brains were averaged with an automated contrast-based method previously described (Friedel et al., 2014; Nieman et al., 2018). Briefly, the individual images first underwent a rigid-body registration where the images are translated and rotated to be in a standard space. In the second step, individual brain images then underwent affine alignment to one target image and an averaged template was generated. The third and last step of the registration involved iterative nonlinear alignment of the individual images to the averaged template to improve the SNR of the average (Avants et al., 2011).

Finally, the average MRI 3D was warped to the AAT using the same warping procedure as the v3D to AAT (Fig. 4). A link to the MRI3D stack is available here (http://repo.mouseimaging.ca/repo/for_svoboda_hhmi/).

### Analyses of electrophysiological features

The extracellular voltage traces were separated into local field potential (LFP) and action potential (AP) bands. The LFP band signal was low-pass filtered at 300 Hz and sampled at 2.5 kHz. AP band was bandpass filtered at 300–5000 Hz and sampled at 30 kHz. For both bands of activity, before analyses, the signals from each channel were first median subtracted to remove any baseline offset from each channel. The signals across the probe then underwent common average referencing where the median across all channels on the probe at each time point was subtracted. Common average referencing is known to remove common noise across the channels on the probe (Ludwig et al., 2009).

Multiunit activity was thresholded from the AP band at −50 μV to register events along electrode sites on the probe. We used Kilosort 2 for spike sorting and registered waveforms and localize them along the probe (Pachitariu et al., 2016). We also used the amplitudes of the spike waveforms and LFP as electrophysiological signatures to identify transitions in brain compartments (Table 2). The visualization of the electrophysiological features and the alignment of the electrodes to ARA compartments were done using the Ephys Atlas GUI (https://github.com/int-brain-lab/iblapps/tree/master/atlaselectrophysiology).

**Table 2:** Table of electrophysiological landmarks

Landmarks (Fig. 7)	Electrophysiological feature
Brain surface/ventricle	Lack of spiking activity and reduced LFP power in saline or CSF compared with the brain
Fiber tract	Reduced activity and smaller spike amplitude compared with cortical regions
CA1 pyramidal cell layer	Higher activity and larger amplitude spikes than other parts of the hippocampus; LFP phase inversion
Hippocampus to thalamus boundary	Higher activity and spike amplitude in thalamic nuclei than hippocampus
Transition from arbor vitae to deep cerebellar nuclei	Higher activity and larger spike amplitude in the deep cerebellar nuclei
Medial superior olive	Sound related-activity in auditory tasks
Gray matter to white matter transition in the cerebellum	Reduced activity and lack of units in the white matter compared with the gray matter
Transition from the vestibular nucleus to other parts of the medulla	Higher spiking activity of neurons in the vestibular nucleus compared with other parts of the medulla

For the groundtruth experiments, we fit a multiterm Gaussian model (fit function in MATLAB) to the eYFP fluorescence and evoked activity along the probe (Figs. 8*E*, 9*E*). The number of Gaussians were specified manually based on the profile of the fluorescence and evoked activity. We compared the corresponding peak locations in fluorescence and evoked activity to quantify the accuracy of the electrode localization procedure (Figs. 10, 12).

## Results

### Overview of the workflows

The goal of the workflows is to localize each electrode (i.e., recording site) on linear arrays and, by extension, the neurons recorded by that electrode, as accurately as possible in a standardized brain coordinate system, the CCF. The CCF corresponds to a high-resolution image volume of averaged brains based on autofluorescence, the AAT, which is used for warping. The AAT is also segmented into brain regions defined by the ARA.

Linear arrays are studded with a regular pattern of electrodes along the shank (Csicsvari et al., 2003; Jun et al., 2017). Localization of electrodes requires reconstruction of the probe track using histologic methods and mapping the probe locations in the CCF. Furthermore, points along the track, such as the probe tip and a subset of electrodes, are localized along the reconstructed track. This is achieved by identifying electrophysiological features that correspond to anatomic landmarks in the ARA, which provides brain region annotations for every location in the CCF. The locations of the remaining electrodes are determined by spatial interpolation according to the known interelectrode spacing. The workflows for localization of electrode locations within the CCF are summarized in Figure 1.

The two workflows share common methods for probe labeling during electrophysiological recording and extraction of the brains. The tissue processing and imaging in the SBF2P workflow is similar to the methods used to create the AAT and CCF. As a result, the features delineating anatomic features are similar and the images can be automatically aligned to the AAT. Moreover, the brains do not require processing postfixation, i.e., clearing (Fig. 1*B*, step 2B), which can shorten the turn-around time by up to 20 d. However, SBF2P is based on complex instrumentation that is not widely available. SPIM requires clearing of the brains, and additional computations to align the 3D images to the AAT, but turnkey SPIM microscopes are widely available. In both workflows, after the probe tracks are mapped to the CCF, we align electrodes to the tracks based on electrophysiological landmarks. As the CCF is nonlinearly distorted compared with the brain *in vivo*, placing other electrodes in the CCF space simply by electrode spacing would introduce errors. Placing electrodes in an undistorted MRI space, which is registered to the CCF, should give more accurate electrode placement. Indeed, we found that aligning electrodes in the MRI brain space can improve the localization accuracy.

### Localization of electrodes in the CCF

**Figure 6.**
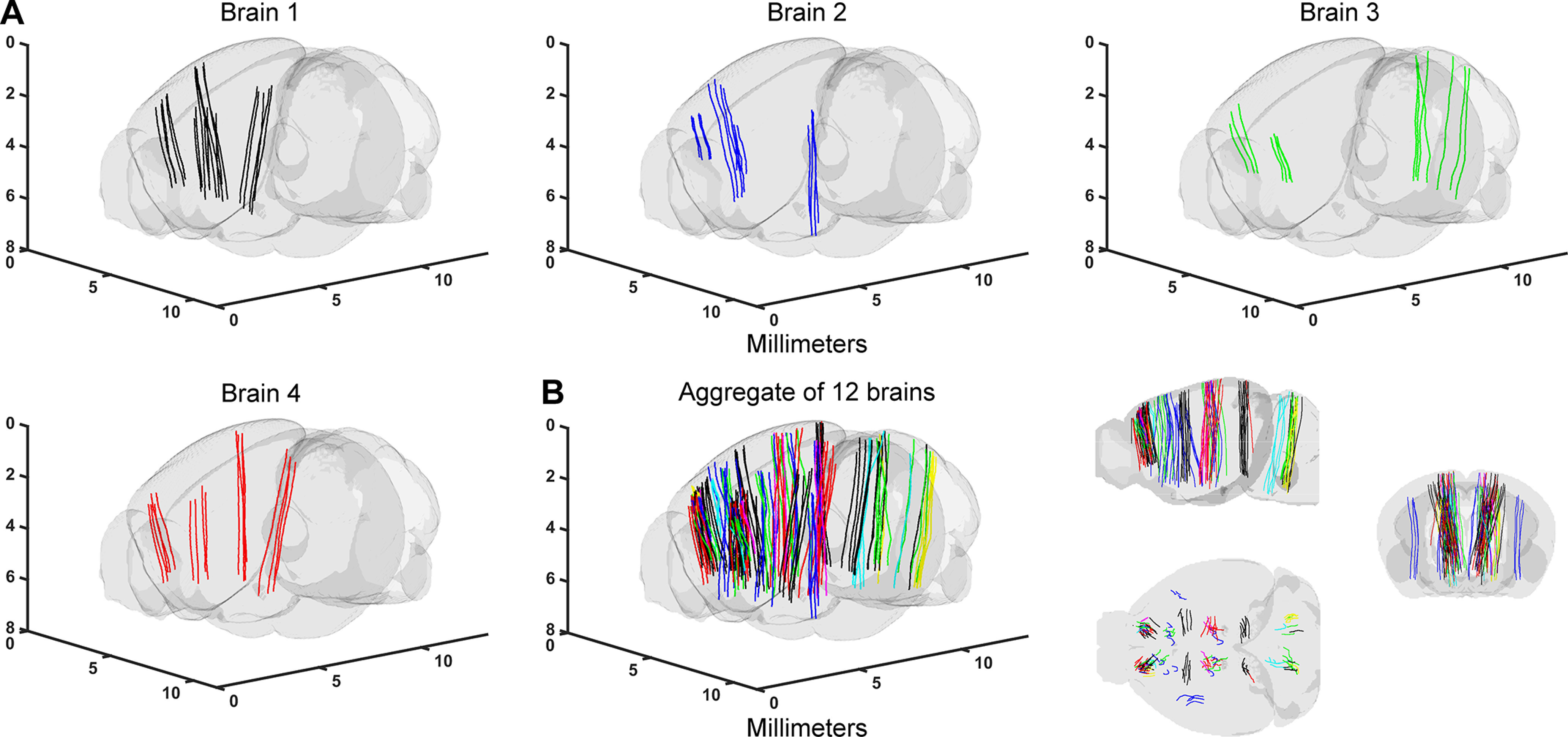
Probe tracks in the CCF for the SPIM workflow. ***A***, Annotated probe tracks in the CCF (4 example brains). Brain 1 corresponds to Figure 3*C*,*D*. ***B***, left, Aggregate of probe tracks in the CCF. Different colors indicate tracks from different brains). Right, Sagittal, horizontal, and coronal views.

We determined the location of each electrode in the CCF using the following steps. (1) To reconstruct each probe track, we manually placed points at ∼0.2 mm intervals on the centerline of the CM-DiI fluorescence in the CCF (∼20 points per penetration; Fig. 1*B*, step 4B). The end of the track was taken to be the point at which DiI fluorescence was no longer visible. A skeleton of the probe track was then determined by linear interpolation between the manually placed points (Figs. 5*B*, 6). (2) We projected the probe track into the MRI3D space using the CCF↔MRI3D transformations (Fig. 1*B*, step 4C). (3) Starting at the end of the probe track, we determined the locations of all electrodes along the track using the known interelectrode spacing (20 μm for pairs of electrodes on the Neuropixels probes). (4) We identified characteristic electrophysiological features along the probe that corresponded to known anatomic landmarks (e.g., consecutive electrodes with little activity that correspond to white matter tracts; Fig. 1*N*, step 5A,B). (5) To improve accuracy, we adjusted the electrode positions to align with these electrophysiological features (Fig. 1*B*, step 5C,D).

We determined the electrode locations within the MRI3D rather than the CCF because the MRI3D is minimally deformed with respect to the *in vivo* brain. We compared the depth of the probe tip in the MRI3D with the depth reading recorded from the micromanipulator. In the CCF the tip location was substantially deeper than the manipulator depth (difference, 1.07 ± 0.37 mm, mean ± SD, 57 penetrations) (at a mean manipulator reading of 3.3 mm), reflecting the fact that the CCF is much enlarged compared with the intact brain (Fig. 4). In the MRI3D space the mean difference was small (0.09 ± 0.26 mm, mean ± SD), reflecting closer resemblance of the MRI3D to the intact brain. The small remaining mean difference between manipulator reading and estimated tip location (0.09 mm, mean) is consistent with dimpling expected at the brain surface after probe insertion (on the order of 0.1 mm; O’Connor et al., 2010). The variability in the difference between manipulator position and tip estimate based on histology was substantial across individual penetrations (0.3 mm, SD, MRI3D). This variability reflects uncertainty in the estimate of tip location based on histology: in some experiments the probe tip was brightly labeled with the dye spreading beyond the probe tip, causing an overestimate of probe tip depth. In other experiments, the tip was dim, resulting in an underestimate of probe tip depth. This uncertainty makes clear why electrophysiological information is critical to estimate the locations of individual electrodes along the probe track.

**Figure 7.**
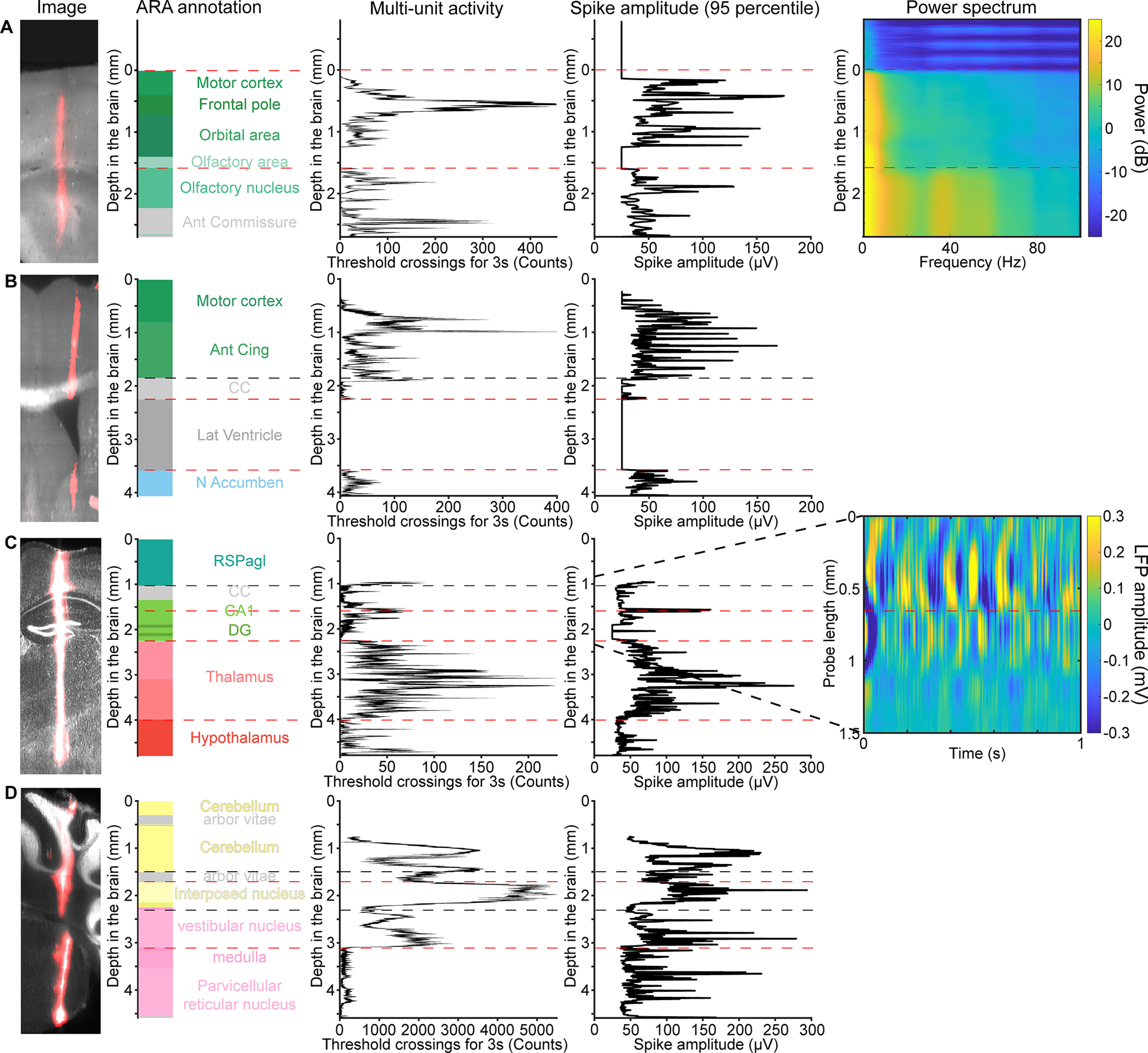
Example electrophysiological landmarks. ***A***, A probe passes through the motor cortex, orbital area, and olfactory nucleus. The transition into the brain is marked by an increase in spiking activity and LFP power. The transition into the olfactory nucleus from the white matter also corresponds to an increase in spiking activity and LFP power. Red dashed lines are the electrophysiological landmarks used to align the electrodes. Black dash lines are the transitions in ARA compartments that are not used for alignment but show good agreement with electrophysiological signatures. ***B***, A probe passes through the cortex, CC, and lateral ventricle. The ventricle lacks neural activity. The CC shows small amplitude axonal spikes. ***C***, A probe passes through the CA1 pyramidal cell layer, which shows up as a narrow band of large-amplitude spikes and a phase inversion of the LFP (right insert, raw LFP amplitude). In addition, the borders of the thalamus are marked by the presence of large amplitude spikes. ***D***, A probe passes through the deep cerebellar nuclei (DCNs) and the medulla. The transition from the DCN to medulla is marked by a dip in spike rate. The upper portion of the medulla corresponds to the vestibular nucleus, which has high spike rates. The arbor vitae (cerebellar white matter), which mostly lacks multiunit activity, is not used as an electrophysiological landmark in the electrode localization process but shows good agreement with the ARA annotation. Extended Data Figure 7-1 shows cross-validation of an example electrophysiological landmark. Extended Data Figure 7-2 shows additional example electrophysiological landmarks.

After the 3D coordinates of all electrode sites were determined (step 3), we projected these coordinates into the CCF. We then determined the anatomic annotation associated with these coordinates using the ARA. We used electrophysiological features recorded on specific electrodes to anchor these electrodes to ARA locations (step 4). Electrophysiological landmarks are anatomic features with recognizable electrophysiological signatures (spiking patterns or LFPs). Examples include: the surface of the brain, with a sharp transition from low amplitude voltage fluctuations outside of the brain to higher amplitude voltage fluctuations and spikes inside the brain (Fig. 7*A*). For recordings where the surface of the brain was not within the 3.84 mm spanned by the first bank of electrode sites of the Neuropixels probe, we recorded from the adjacent bank of electrodes to localize the first electrode in the brain (Extended Data Fig. 7-2*A*). Additional electrophysiological landmarks include: white matter, such as the CC, which shows mainly small amplitude axonal spikes compared with the larger spikes in the neighboring gray matter (Fig. 7*B*); ventricles, with no spikes and low amplitude voltage fluctuations (Fig. 7*B*); CA1 layer of the hippocampus, with large amplitude spikes and a phase inversion of the LFP (Buzsáki et al., 2012; Fig. 7*C*); hippocampus, thalamus border, with low spike rates in the hippocampus, and large amplitude spikes and high spike rates in the thalamus (Fig. 7*C*); arbor vitae, deep cerebellar nuclei border, with higher spike rates and larger amplitude spikes in the deep cerebellar nuclei (the transition in spike rate was not observed in some recordings; Fig. 7*D*); white-matter. 2 t’s and gray-matter border in the cerebellum, with lower spike rate and reduced number of units in the white matter compared with the gray matter (Fig. 7*D*); vestibular nucleus in the medulla often has higher spike rate than other parts of the medulla (Extended Data Fig. 7-2*C*).

10.1523/ENEURO.0241-21.2021.f7-1 Extended Data Figure 7-1 Cross-validation of the example electrophysiological landmark. ***A***, The ARA annotation along with the LFP power, unit spike rate and unit amplitude across an example probe penetration. The increase in LFP power is an electrophysiological landmark used to localize the electrode at the surface of the brain (red dashed line). The interpolated electrodes beneath the landmark show good agreement at the transition between L1 and L2/3 of the motor cortex (black dashed line), where there is a lack of units at L1. Each dot represents a unit and the line indicates the moving average. ***B***, After aligning the electrodes using the electrophysiological landmark at the surface of the brain. The landmark is cross-validated by aggregating the sites around the transition of L1 to L2/3 for multiple probes. Each color represents the moving average from one probe insertion. Red dash lines indicate the surface of the brain used for alignment. Black dashed line is the transition between L1 and L2/3 of the motor cortex. L1 lacks units above 80 μV. Download Figure 7-1, TIF file.

10.1523/ENEURO.0241-21.2021.f7-2 Extended Data Figure 7-2 Additional example electrophysiological landmarks. ***A***, For all penetrations covering a distance longer than the bottom bank of the probe (3.84 mm for Neuropixels 1.0 probes). We can estimate surface by recording from a bank of electrodes above the bottom bank. code_cache/lfpSurface at master · hanhou/code_cache (https://github.com/). ***B***, Example probe insertion passing through the fourth ventricle. ***C***, A probe passing through the vestibular nucleus. The vestibular nucleus in the medulla often has higher firing rate units than other parts of the medulla. Download Figure 7-2, TIF file.

The electrophysiological landmarks were used to localize a subset of electrodes to ARA compartments or transitions between compartments (Fig. 7, red dashed lines, step 5). For interpolation and extrapolation of all electrode sites on the probe, two or more electrophysiological landmarks need to be localized to electrodes. The interelectrode distances in between two electrophysiological landmarks are scaled linearly using a common scaling factor in the MRI3D or CCF. The interelectrode distance above the first electrophysiological landmark or the interelectrode distance below the last electrophysiological landmark are extrapolated using the nearest scaling factor. When only one electrophysiological landmark is available, we anchor the corresponding electrodes to one landmark, and use the scaling factor from other penetrations in the same brain to extrapolate the other electrodes.

Several analyses support the validity of our workflows for electrode localization. First, the extrapolated electrode tip locations corresponded to electrode depths derived from manipulator readings (difference, 0.08 ± 0.11 mm, mean ± SD). Second, we cross-validated the electrophysiological landmark at the surface of the brain (i.e., an increase in LFP power inside the brain) by analyzing other electrophysiological signatures beneath the brain surface. Layer (L)1 of the cortex contains only a low density of GABAergic interneurons and few detected spikes (Douglas and Martin, 2004; Lefort and Petersen, 2017). The transition between L1 and L2/3 of the motor cortex is characterized by the appearance of units with amplitude above 80 μV, corresponding to pyramidal neurons (Extended Data Fig. 7-1). These results show the electrodes around the surface of the brain accurately reflect the expected differences in electrophysiological features. Also, the transition between different brain regions exhibits changes in electrophysiological features that could be used for the localization of electrodes.

### Groundtruth experiment

We next developed an independent set of experiments to quantify the accuracy of the workflows for electrode localization. We performed experiments in wild-type C57BL/6J mice. Neurons in the left anterior lateral motor cortex (2.5 mm anterior of bregma, −1.5 mm lateral) were transduced with AAV virus expressing ChR2-eYFP (Guo et al., 2014b; Li et al., 2015). We recorded from downstream brain regions that contained small axonal projections expressing ChR2-eYFP, including a subset of the locations we used as electrophysiological landmarks (based on neural activity outside of photostimulation). Photostimulation of these axons produces phasic neural activity with short latencies on electrodes near the ChR2-eYFP-expressing axons.

We used this activity to confirm that we have correctly identified key electrophysiological landmarks, such as the white matter. More importantly, the intersection of the eYFP signal in 3D volumes and the probe track provides an independent confirmation of the location of the electrophysiological landmarks.

**Figure 8.**
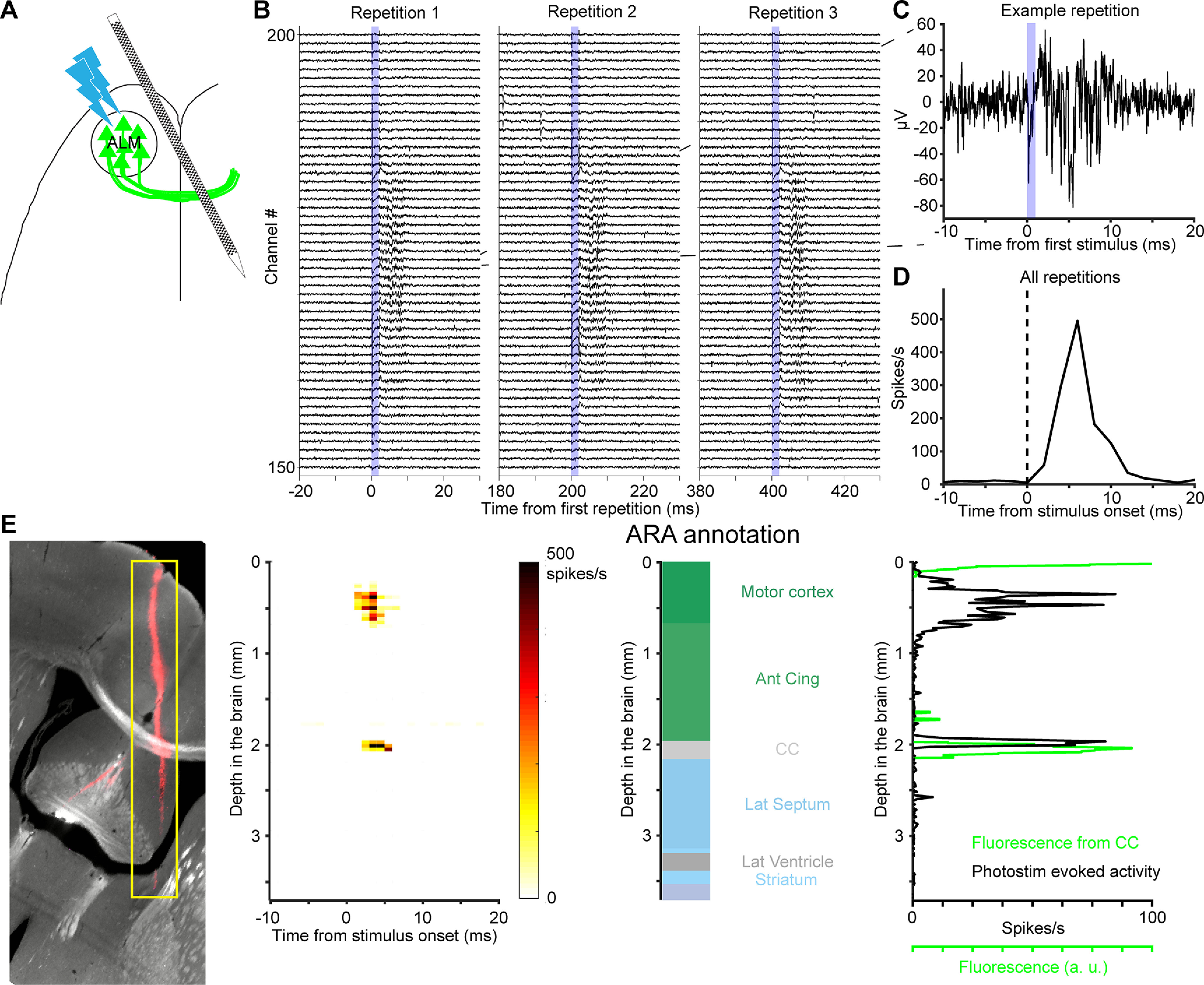
Comparing localization of CC using fluorescence and photostimulation-evoked activity. ***A***, Schematic of the experiment. ChR2-eYFP was expressed in ALM neurons. A photostimulus was applied over ALM to elicit spikes, while recording from contralateral ALM axons. ***B***, Example voltage traces show evoked multiunit activity in the CC during three successive photostimuli. Voltage traces were from 50 electrodes around the CC. The blue shading indicates the 2-ms stimulus pulse duration. ***C***, Voltage trace from one electrode in response to one photostimulus. ***D***, Peristimulus time histogram of multiunit events (averaged over 300 repetitions for the channel in ***C***). ***E***, left, A coronal section showing fluorescence from ChR2-eYFP (light gray) and CM-DiI (red) labeled probe track. The evoked multiunit activity across the probe is localized to 5 ms after the stimulus onset. Middle, ARA annotation along the probe. Right, Intensity of ChR2-eYFP fluorescence (green) and evoked activity (black) along the localized electrode in the CCF. Importantly, the CC was not used as an electrophysiological landmark in the electrode localization process.

ALM axons from the left hemisphere cross the CC into homotypic ALM in the right hemisphere (Li et al., 2015). Small multiunits events reflecting axonal spikes were visible in extracellular recording (Fig. 8*B*). In response to short optogenetic stimuli (duration 2 ms) we detected a phasic increase in multiunit activity with short latency (2 ms; Fig. 8*C*).

**Figure 9.**
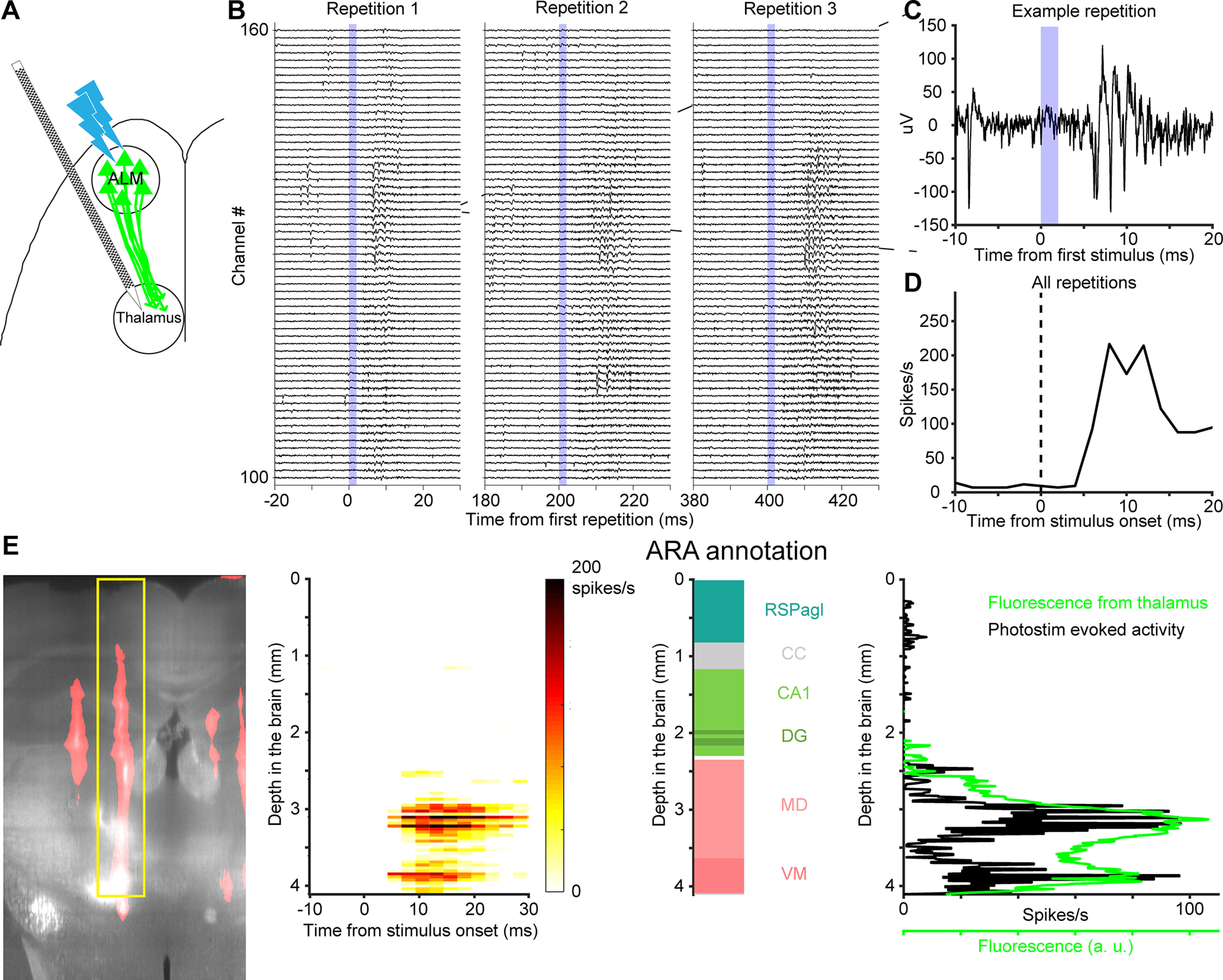
Comparing localization of thalamus using fluorescence and photostimulation-evoked activity. ***A***, Schematic of the experiment. Activity was recorded in the thalamus. ***B***, Example voltage traces show multiunit activity in the thalamus. Same as Figure 8*B* but for 60 electrodes in the thalamus. ***C***, Voltage trace from one electrode in response to one photostimulus. ***D***, PSTH of multiunit events. Averaged over 300 photostimulus repetitions for the channel in ***C***. ***E***, left, A coronal section showing fluorescence from ChR2-eYFP (light gray) and CM-DiI (red) labeled probe track. The evoked multiunit activity across the probe is between 5 and 20 ms after the stimulus onset. Middle, ARA annotation along the probe. Right, Intensity of ChR2-eYFP fluorescence (green) and evoked activity (black) along the probe in the CCF. Importantly, the medial dorsal and ventral medial nuclei of the thalamus were not used as electrophysiological landmarks for electrode localization.

ChR2-eYFP-expressing axons also project to the mediodorsal and ventromedial nucleus of the thalamus (MD and VM; Guo et al., 2017). Photostimulation of ALM evoke postsynaptic responses in the MD and VM neurons (Fig. 9*B*). The responses in the MD and VM are of longer latency (5 ms; Fig. 9*C*), consistent with the length of synaptic delay.

**Figure 10.**
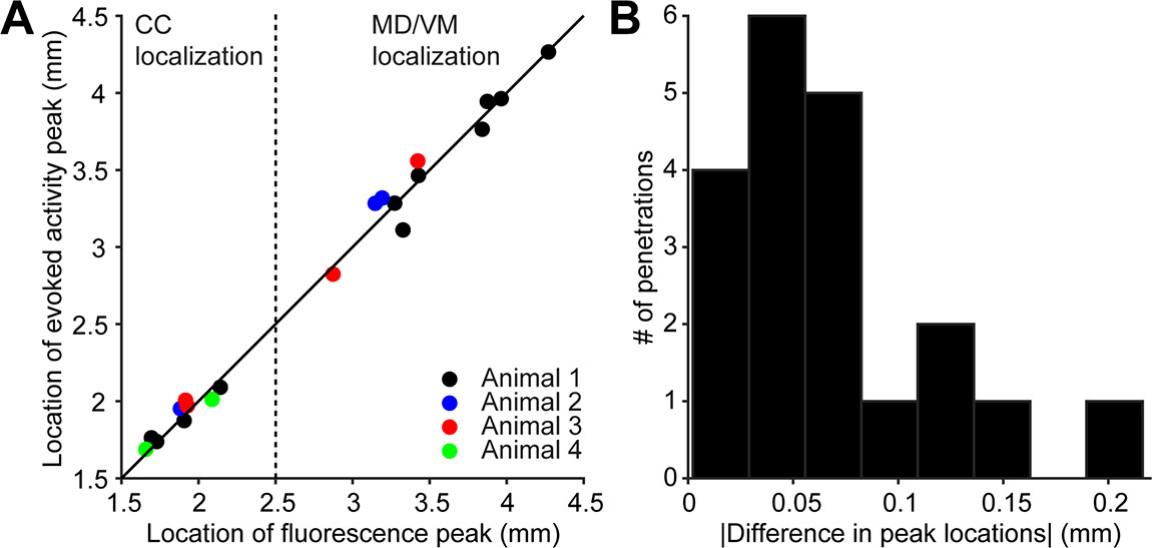
Accuracy of electrode localization assessed for the SPIM workflow. ***A***, The peak locations of evoked activity and eYFP fluorescence were estimated using a Gaussian fit (4 mice, 17 penetrations). ***B***, The distance between the peak locations of evoked activity and eYFP fluorescence. Absolute value of the difference in peak locations was used to quantify electrode localization accuracy (0.069 ± 0.054 mm, mean ± SD).

**Figure 11.**
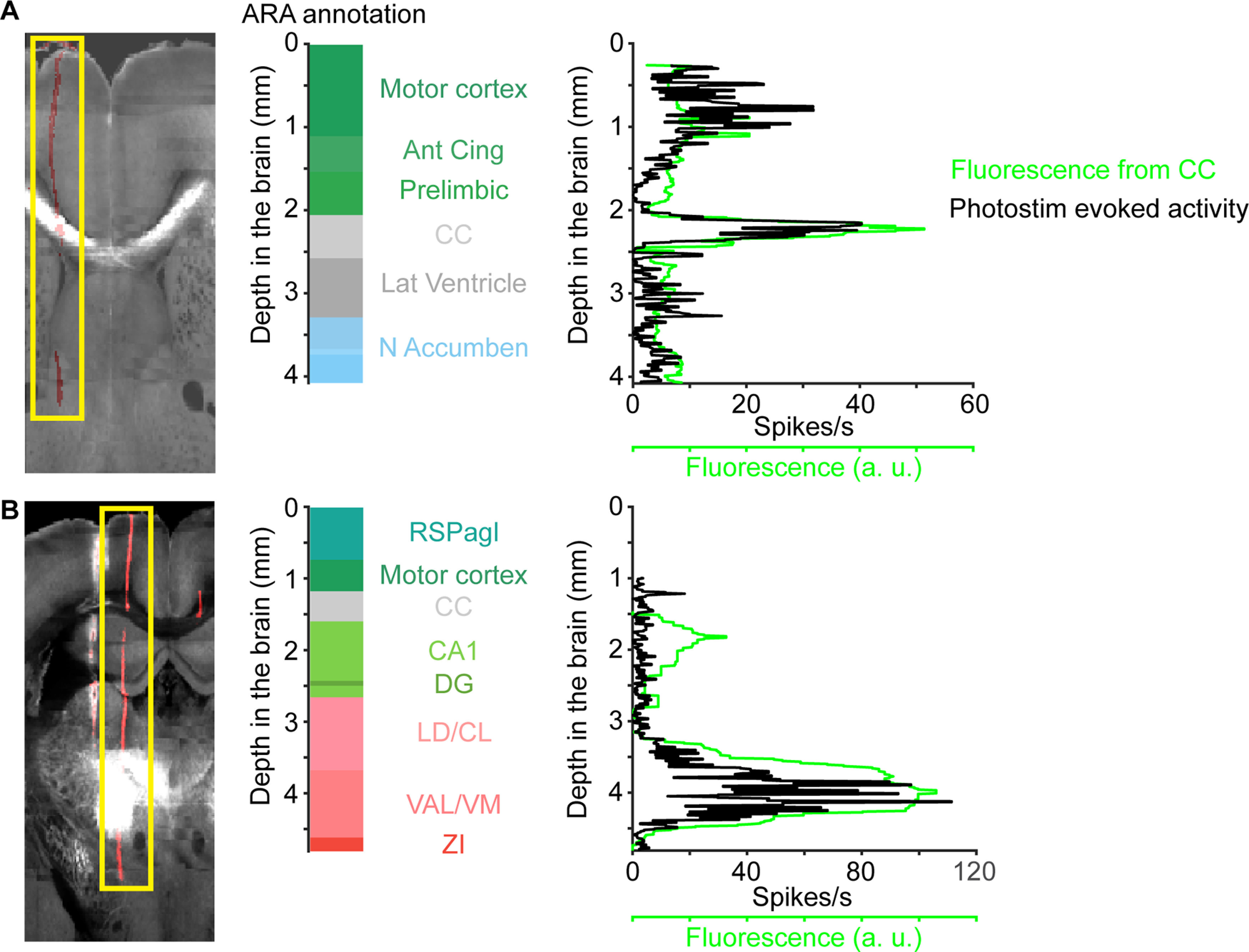
Comparing fluorescence and photostimulation-evoked activity from the SBF2P workflow. ***A***, ***B***, left, A coronal section showing fluorescence from ChR2-eYFP (light gray) and CM-DiI (red) labeled probe track. Middle, ARA annotation along the probe. Right, Intensity of ChR2-eYFP fluorescence (green) and evoked activity (black) along the probe in the CCF.

**Figure 12.**
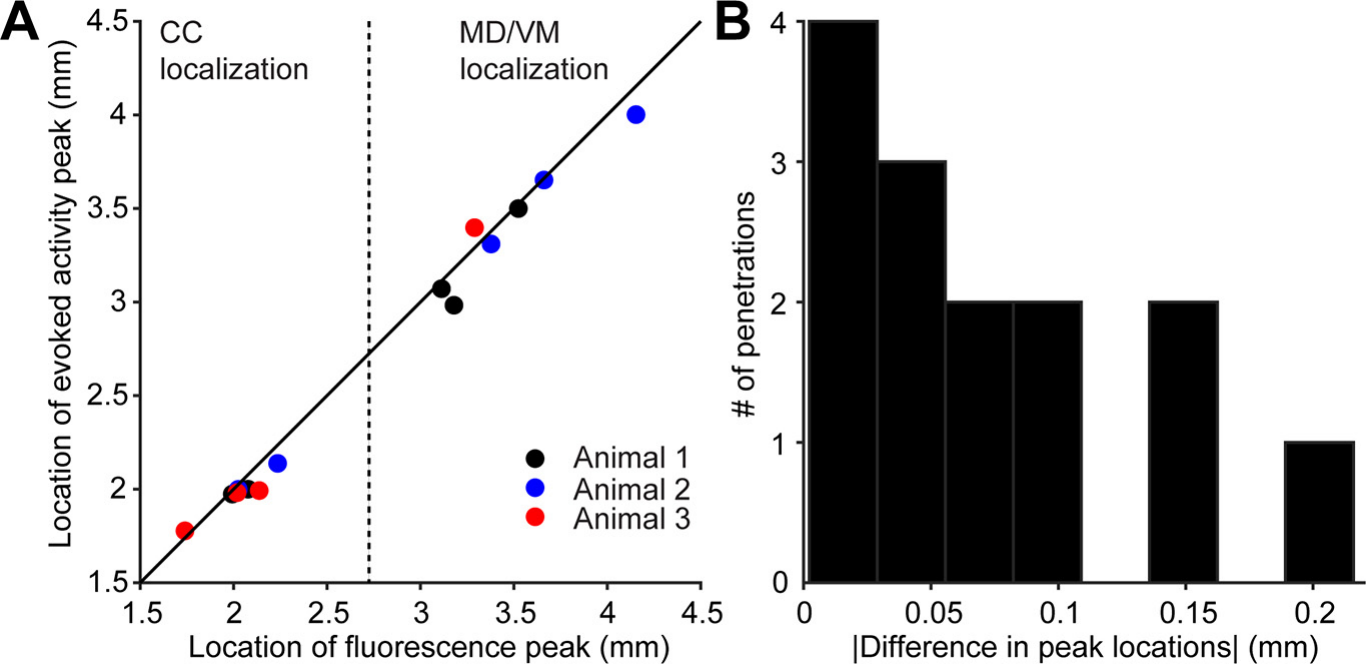
Accuracy of electrode localization assessed for the SBF2P workflow. ***A***, The peak locations of evoked activity and eYFP fluorescence were estimated using a Gaussian fit (3 mice, 14 penetrations). ***B***, The distance between the peak locations of evoked activity and eYFP fluorescence. Similar accuracy was observed for the SBF2P workflow (0.074 ± 0.058 mm, mean ± SD).

The evoked activity confirmed the electrode placement using electrophysiological landmarks. We localized each electrode in v3D and CCF, in which the eYFP fluorescence indicates the ChR2 expression. In both workflows, the profile of the evoked activity on the electrodes resembled the profile of the eYFP fluorescence (Figs. 8*E*, 9*E*, 11*A*,*B*). Quantifying the peak locations of the fluorescence and evoked activity gave us a quantitative description of the accuracy of the alignment procedure (Figs. 10*A*,*B*, 12*A*,*B*; Materials and Methods).

In the SPIM workflow, comparison of the peak locations of fluorescence and photostimulation-evoked activity yields an accuracy of 0.069 ± 0.054 mm (mean ± SD; Fig. 10*A*,*B*). We also evaluated the impact of using the MRI3D for electrode placement. We directly placed electrodes in CCF (without MRI3D), using linear interpolation between electrophysiological landmarks. Direct placement of electrodes in CCF was less accurate compared with placements in the MRI3D (0.125 ± 0.063 mm, mean ± SD; *p *=* *0.003, two-tailed paired *t* test), justifying the use of the MRI3D.

In a separate group of mice, we tested the accuracy of electrode localization in the SBF2P workflow (for comparison of the workflows, see Fig. 1*B*). We performed the electrode localization in the registered brain. Electrodes were localized to the ARA compartments using electrophysiological landmarks as described above. We found similar accuracy in these brains (0.074 ± 0.058 mm, mean ± SD) that was comparable to the SPIM workflow (Fig. 12*A*,*B*). Placement of electrodes in the MRI3D instead of the CCF did not improve the localization accuracy for SBF2P images that were automatically registered (0.075 ± 0.069 mm, mean ± SD; *p *=* *0.95, two-tailed paired *t* test). These results show that the comparison between fluorescence and photostimulation-evoked activity is a robust method to assess the accuracy of electrode localization across different imaging and registration methods.

## Discussion

We describe two workflows to localize electrodes along a linear probe in a standardized mouse brain coordinate system. During recordings, probe tracks were marked with fluorescent dye that persisted in the tissue across multiple experiments spanning weeks. After the experiments, the brain volume and probe tracks were imaged *ex vivo* using either a combination of tissue clearing and SPIM or SBF2P imaging. The imaged volume was computationally warped to the CCF and the probe tracks reconstructed. Individual electrodes were localized along the probe track based on electrophysiological signatures and other electrodes were assigned by interpolation and extrapolation. Groundtruth experiments indicate that both workflows have a mean accuracy of around 0.07 mm for localizing electrodes in the CCF.

Neuropixels probes (Jun et al., 2017; Steinmetz et al., 2021) and other large linear probes (Berényi et al., 2014; Shobe et al., 2015; Raducanu et al., 2016; Fiáth et al., 2018) sample activity across multiple brain regions. Interpretation of neural activity in terms of neural circuits relies on accurate localization of individual electrodes and thereby the recorded neurons. Several workflows have been described (Allen et al., 2019; Steinmetz et al., 2019; Siegle et al., 2021), but groundtruth experiments assessing the accuracy of electrode localization are lacking. We took advantage of ChR2-EYFP expression in focused axonal projections. We then compared the locations of ChR2-EYFP expression, as judged by fluorescence, with light-evoked activity, measured with electrodes that were registered to the CCF. This method can be applied to assess the accuracy of other electrode localization workflows using different types of linear probes, different 3D imaging methods, and possibly in different species.

The CCF is distorted compared with the intact brain: the brain is enlarged and locally sheared, especially around the ventricles. We obtained an in-skull MRI image stack (MRI3D), which prevented enlargement and local distortion of the brain. Mice were matched by strain, age, sex and experimental condition (water restriction). The MRI3D resembled the *in vivo* conditions of the recorded brains (de Guzman et al., 2016). The placement of electrodes in the MRI3D image space was more similar to the electrode spacing in the intact brain, thereby, leading to more accurate estimates when extrapolating electrode locations based on distant electrophysiological landmarks or absence of electrophysiological landmarks. Without using electrophysiological landmarks, the estimated tip location in the CCF was on average 1 mm longer than the micromanipulator reading compared with 0.1 mm longer when estimated in the MRI3D. In the groundtruth experiments, where electrophysiological landmarks were used, the inclusion of the MRI3D still produced a consistent improvement in the accuracy of electrode localization for the SPIM workflow (average error from 0.12 to 0.07 mm) by allowing scaling in a space closer to the intact brain. However, no improvement was detected for the SBF2P workflow. This difference could be because of the small sample size.

Brain shapes differ across different mouse strains and also depend on the sex and age of the animals. For example, we note that the thickness of the motor cortex of our laboratory mice were ∼10% smaller than C57Bl/6J mice previously imaged under the same conditions using MRI (Dorr et al., 2008; Steadman et al., 2014). Using a workflow relying on MRI3D may require acquisition of additional MRI volumes for different mouse strains.

We detect the centroids of fluorescence and light-evoked activity, and use the difference as a measure of accuracy of localization. This is likely an upper bound on the error in localization. The spread of the axonal projections limits the resolution of this method. In addition, photostimulation-evoked activity may not always exactly correspond to axonal fluorescence. These factors create a potential source of mismatch when comparing fluorescence with photostimulation-evoked activity. Projections to the thalamus are broader than the axon bundles in the CC (Figs. 8, 9, 11). Seven out of eight of the penetrations with localization error larger than 0.1 mm were in the thalamus (Figs. 10, 12). Moreover, the less accurately localized penetrations are distributed across individual brains, making it unlikely that the errors are caused by inaccuracy in registering image volumes. These factors suggest that the outliers are caused by systematic errors in the groundtruth experiment and that the localization accuracy is likely better than 0.07 mm on average.

Histologic methods by themselves were not sufficient to localize electrodes (errors up to 0.3 mm, SD). Electrophysiological signatures are necessary for anchoring sites along the probe, followed by interpolation and extrapolation outside of the electrophysiological landmarks (Buzsáki et al., 2012; Jun et al., 2017; Senzai et al., 2019; Steinmetz et al., 2019; Peters et al., 2021; Siegle et al., 2021). Our work further extends this concept to multiple brain regions and provides validation of the electrophysiological landmarks through groundtruth experiments.

One potential limitation when applying the electrode localization workflow with SPIM at large scale is the manual landmark placement for warping individual v3Ds to the CCF. The difference in fluorescence between the v3D and the CCF in the SPIM workflow prevents the use of standard fully automated alignment methods (Klein et al., 2010; Kuan et al., 2015). This issue could be overcome by constructing an in-house template for the v3D brains using the same tissue preparation and imaging conditions. The template can be carefully warped to the CCF using the manual procedures described. Each individual v3D can then be warped to the template automatically instead of warping to the CCF.

Another manual step is the identification of electrophysiological landmarks for interpolation of electrode sites. The electrophysiological landmarks identified here tend to be robust in the regions of interest (cross-validation of electrophysiological landmarks in Extended Data Fig. 7-1 and small localization error in the motor cortex and thalamus penetrations). Probe tracks traversing through the same boundaries between brain regions will have the same electrophysiological landmarks. Localizing electrophysiological landmarks is done by matching electrophysiological (e.g., spike rate) and anatomic features (Table 2). This step requires knowledge of the underlying anatomy and physiology. As data from large-scale recording experiments become available, detailed analysis of electrical activity in different brain regions might allow automated procedures. In the future, automating (1) warping of v3D to template brains, (2) reconstruction of probe tracks, and (3) anchoring of electrodes using defined electrophysiological landmarks will greatly accelerate the electrode localization workflows.

## Synthesis

Reviewing Editor: Laura Bradfield, University of Technology Sydney Decisions are customarily a result of the Reviewing Editor and the peer reviewers coming together and discussing their recommendations until a consensus is reached. When revisions are invited, a fact-based synthesis statement explaining their decision and outlining what is needed to prepare a revision will be listed below. The following reviewer(s) agreed to reveal their identity: Daniel Denman.

Thank you for submitting your work to eNeuro, both reviewers felt that the manuscript offered a significant advance by providing comprehensive methods of localisation for silicon probes in mouse brains.

Reviewer #2 had some specific comments to be addressed below. In addition to these comments, it was also suggested that Figures 2-6 and some of the supplemental figures could be compressed into fewer figures to increase clarity and impact of the manuscript.

The specific reviews are as follows:

Reviewer #1

The manuscript provides a comprehensive overview of two analysis pipelines to visualize silicone probe placement in mice brains. The workflow is made very clear. Overall I think this is a timely article that will benefit the field. Minor concerns and comments below.

I found Figure 1 and Supp Figure 1-1 to be equally helpful. I realize there are already quite a few figures in the manuscript, but bringing Supp Figure 1-1 into the main manuscript should be considered.

I can’t tell for sure, but Supplemental Figure 4.3 appears to use the jet colormap. If so, the authors should consider using colormaps that are perceptually uniform. Jet has known problems (https://www.nature.com/articles/s41467-020-19160-7).

Not a concern, but a comment. I assume the authors provided detailed of workflows for localization techniques utilizing tissue clearing and SPIM because these are the methods in place in their labs. This is entirely reasonable. However, each of these methods require considerable resources in some capacity. In order for more labs to utilize and embrace high-density recordings, like Neuropixels, more cost effective methods will need to be developed. I realize this is beyond the scope of the current manuscript, just a thought for a need that will arise.

Reviewer #2

The authors present a comprehensive workflow for localizing high-density linear probes, and particularly Neuropixels, in a standardized mouse brain coordinate system. While such workflows for registration of probe tracks and the requisite warping, this work presents novel adjustment using electrophysiological landmarks and evaluation of localization accuracy using ontogenetically evoked activity and the corresponding opsin fluorescence. This assessment of accuracy is of great value and I think should be more forefronted. I also think, pending some clarification, the use of electrophysiological landmarks to assist the warping is of great potential value to users of these electrodes.

My major questions:

1. While the distortions of brains that make up the CCF is clear, and that the MRI volume from these mice in which the recording were made is more accurate, it is not clear if the intention of this paper is to generally provide a(nother) standard MRI brain atlas for more accurate registration of electrodes in a less distorted way than the AAT (and therefore with CCG annotations) or more to evaluate the accuracy just for this work. I would suggest that first three sections could be compressed and clarified. Given that Neuropixels recording, the imaging methods, and three dimensional templates are all methods decribed elsewhere, these feel more appropriate in the methods. If I there is another point of these sections in the resuts, it should be clearer.

2. Was electrophysiological scaling more or less accurate for particular areas of the brain? Are the examples listed on lines 553 - 563 and Table 2 comprehensive? More complete details on this procedure would greatly strengthen the entire paper.

3. Was electrophysiological scaling applied to the groundtruth experiment? Should this scaling not make the CCF more accurate without having to warp that to an MRI volume?

Minor comments:

The juxtaposition of these lines is quite confusing to me:

Line 446: using high-resolution MRI (Kovacević et al., 2005; Spencer Noakes et al., 2017). 446 These image volumes are consistent across individual mice (Supplemental Figure 4.2) (Lerch et 447 al., 2008)

Line 451: Depending on strain, sex, and age, mice show considerable variation in brain size and cortical 453 thickness (approximately 15%) (Chen et al., 2006; Qiu et al., 2018; Scholz et al., 2016), as 454 reflected in existing mouse MRI atlases (Dorr et al., 2008; Johnson et al., 2010; Steadman et 455 al., 2014).

Line 541: agreed, this is a strength and could bee emphasized more.

The authors provide a link to 3D brain volumes, and i think they would be useful to be made available alongside any publication.

A big picture comment: the abstract rightly highlights the evaluation of electrode accuracy (as opposed to creating a new one). I think the introduction and results could really be focused on these experiments. I think less could be made of the two workflows in the results - differences should be pointed out, but it feels like it distracts from the main point of what was presented.

## Author Response

Reviewer #1

The manuscript provides a comprehensive overview of two analysis pipelines to visualize silicone probe placement in mice brains. The workflow is made very clear. Overall I think this is a timely article that will benefit the field. Minor concerns and comments below.

I found Figure 1 and Supp Figure 1-1 to be equally helpful. I realize there are already quite a few figures in the manuscript, but bringing Supp Figure 1-1 into the main manuscript should be considered.

Thank you for the suggestion. We brought Supp Figure 1-1 to the main manuscript as Figure 1B.

I can’t tell for sure, but Supplemental Figure 4.3 appears to use the jet colormap. If so, the authors should consider using colormaps that are perceptually uniform. Jet has known problems (https://www.nature.com/articles/s41467-020-19160-7).

Supplemental Figure 4.3 indeed used the jet colormap. In the revised manuscript. We used the perceptually uniform batlow colormap as suggested by the reviewer.

Not a concern, but a comment. I assume the authors provided detailed of workflows for localization techniques utilizing tissue clearing and SPIM because these are the methods in place in their labs. This is entirely reasonable. However, each of these methods require considerable resources in some capacity. In order for more labs to utilize and embrace high-density recordings, like Neuropixels, more cost effective methods will need to be developed. I realize this is beyond the scope of the current manuscript, just a thought for a need that will arise.

Tissue clearing with SPIM is routinely used in our institute. The reagents for brain clearing are commercially available and SPIM microscopes are available at the imaging center of many universities. The total cost is on the order of hundreds of dollars per brain. If we consider the labor and equipment involved in other methods of imaging probe tracks, i.e., sectioning the brain into 50 μm slices and imaging each slice, the cost of brain clearing and SPIM makes a lot of sense in our opinion.

Reviewer #2

The authors present a comprehensive workflow for localizing high-density linear probes, and particularly Neuropixels, in a standardized mouse brain coordinate system. While such workflows for registration of probe tracks and the requisite warping, this work presents novel adjustment using electrophysiological landmarks and evaluation of localization accuracy using ontogenetically evoked activity and the corresponding opsin fluorescence. This assessment of accuracy is of great value and I think should be more forefronted. I also think, pending some clarification, the use of electrophysiological landmarks to assist the warping is of great potential value to users of these electrodes.

My major questions:

1. While the distortions of brains that make up the CCF is clear, and that the MRI volume from these mice in which the recording were made is more accurate, it is not clear if the intention of this paper is to generally provide a(nother) standard MRI brain atlas for more accurate registration of electrodes in a less distorted way than the AAT (and therefore with CCG annotations) or more to evaluate the accuracy just for this work. I would suggest that first three sections could be compressed and clarified. Given that Neuropixels recording, the imaging methods, and three dimensional templates are all methods decribed elsewhere, these feel more appropriate in the methods. If I there is another point of these sections in the resuts, it should be clearer.

The intention of the MRI volume was to evaluate the accuracy of our work. Indeed we found substantial strain, sex, and age differences that may make our and other MRI volumes incompatible with other studies (Line 740-744): “Brain shapes differ across different mouse strains and also depend on the sex and age of the animals. For example, we note that the thickness of the motor cortex of our laboratory mice was approximately 10% smaller than C57Bl/6J mice previously imaged under the same conditions (Dorr et al., 2008; Steadman et al., 2014). Using the MRI3D workflow introduced here may require acquisition of additional MRI volumes for different mouse strains.”

In the revised manuscript, we moved Neuropixels recording, the imaging method and three-dimensional templates from the results to the methods. This revision shortened the manuscript considerably and made it clearer.

2. Was electrophysiological scaling more or less accurate for particular areas of the brain? Are the examples listed on lines 553 - 563 and Table 2 comprehensive? More complete details on this procedure would greatly strengthen the entire paper.

The accuracy of electrophysiological scaling depends on the accuracy of localizing electrophysiological landmarks. In areas of the brain where we are less sure of the landmarks, the alignment of electrodes is expected to be less accurate. The examples listed on lines 553 - 563 and Table 2 are not comprehensive, and the field is discovering new electrophysiological landmarks as recordings are performed in more brain areas. In the revised manuscript, we added more examples (Table 2) to provide complete details on this procedure. Additional examples such as the estimation of the surface of the brain when the probe insertion depth is greater than 3.84mm, fourth ventricle, and vestibular nucleus etc. are now included in the revised manuscript. We added the examples as Supplementary Figure 7.2.

3. Was electrophysiological scaling applied to the groundtruth experiment? Should this scaling not make the CCF more accurate without having to warp that to an MRI volume?

Electrophysiological scaling was applied to the groundtruth experiment. This scaling is expected to make electrode alignment more accurate compared to not using electrophysiological scaling at all. The error was 0.12 mm for the SPIM pipeline without having to warp to an MRI volume. Warping to the MRI volume reduced the error from 0.12 mm to 0.07 mm by allowing nonlinear scaling between the electrophysiological landmarks based on the warp. I.e. electrophysiological scaling in the CCF without warping to the MRI volume is only linear. We decided to highlight this difference in the manuscript since it highlights an important conceptual issue. As the reviewer also notes, when electrophysiological landmarks are ambiguous or not present, warping to the MRI volume can improve electrode alignment. We clarified this point in the discussion of the revised manuscript. Line 727-738,

"The MRI3D resembles the in vivo conditions of the recorded brains (de Guzman et al., 2016). The placement of electrodes in the MRI3D image space was thus more similar to the electrode spacing in the intact brain, thereby leading to more accurate estimates when extrapolating electrode locations based on distant electrophysiological landmarks or absence of electrophysiological landmarks. Without using electrophysiological landmarks, the estimated tip location in the CCF was on average 1 mm deeper than the micromanipulator reading compared to 0.1 mm deeper when estimated in the MRI3D. In the groundtruth experiments, where electrophysiological landmarks were used, the inclusion of the MRI3D still produced a consistent improvement in the accuracy of electrode localization for the SPIM workflow (average error from 0.12 mm to 0.07 mm) by allowing scaling in a space closer to the intact brain. However, no improvement was detected for the SBF2P workflow. This difference could be because of the small sample size.”

Minor comments:

The juxtaposition of these lines is quite confusing to me:

Line 446: using high-resolution MRI (Kovacević et al., 2005; Spencer Noakes et al., 2017). 446 These image volumes are consistent across individual mice (Supplemental Figure 4.2) (Lerch et 447 al., 2008)

Line 451: Depending on strain, sex, and age, mice show considerable variation in brain size and cortical 453 thickness (approximately 15%) (Chen et al., 2006; Qiu et al., 2018; Scholz et al., 2016), as 454 reflected in existing mouse MRI atlases (Dorr et al., 2008; Johnson et al., 2010; Steadman et 455 al., 2014).

We apologize for the confusion. In the revised manuscript, we moved the references to the end of the sentences to avoid confusion. In the new line 293,

"These image volumes are consistent across individual mice (Supplemental Figure 4.2) and are less distorted compared to brains after extraction from the skull (de Guzman et al., 2016; Ma et al., 2008; Lerch et al., 2008).”

and line 299,

Depending on strain, sex, and age, mice show considerable variation in brain size and cortical thickness (approximately 15%), as reflected in existing mouse MRI atlases (Chen et al., 2006; Dorr et al., 2008; Qiu et al., 2018; Johnson et al., 2010; Scholz et al., 2016; Steadman et al., 2014).

Line 541: agreed, this is a strength and could bee emphasized more.

We agree with the reviewer’s suggestion. In the revised manuscript, we emphasized the use of electrophysiological landmarks. The results section only focuses on the localization of electrodes using electrophysiological landmarks and the groundtruth experiments. We moved Neuropixels recording, the imaging methods, and the three-dimensional templates sections to the methods.

The authors provide a link to 3D brain volumes, and i think they would be useful to be made available alongside any publication.

The 3D brain volumes are made available in the revised submission as Extended Data 3.1.

A big picture comment: the abstract rightly highlights the evaluation of electrode accuracy (as opposed to creating a new one). I think the introduction and results could really be focused on these experiments. I think less could be made of the two workflows in the results - differences should be pointed out, but it feels like it distracts from the main point of what was presented.

We shifted the focus of the introduction and results to focus on the experiments evaluating the accuracy of electrode localization. In the revised manuscript, we moved the Neuropixels recording, the imaging methods and the three-dimensional templates from the results to the methods, this shifts the focus of the results to the differences between the two workflows and the groundtruth experiments.
