# Supplementary figures and images for: Accurate Localization of Linear Probe Electrode Arrays across Multiple Brains
Source: eNeuro. 2021 Nov 10;8(6):ENEURO.0241-21.2021. doi: 10.1523/ENEURO.0241-21.2021 (PMC8597948; doi:10.1523/ENEURO.0241-21.2021)

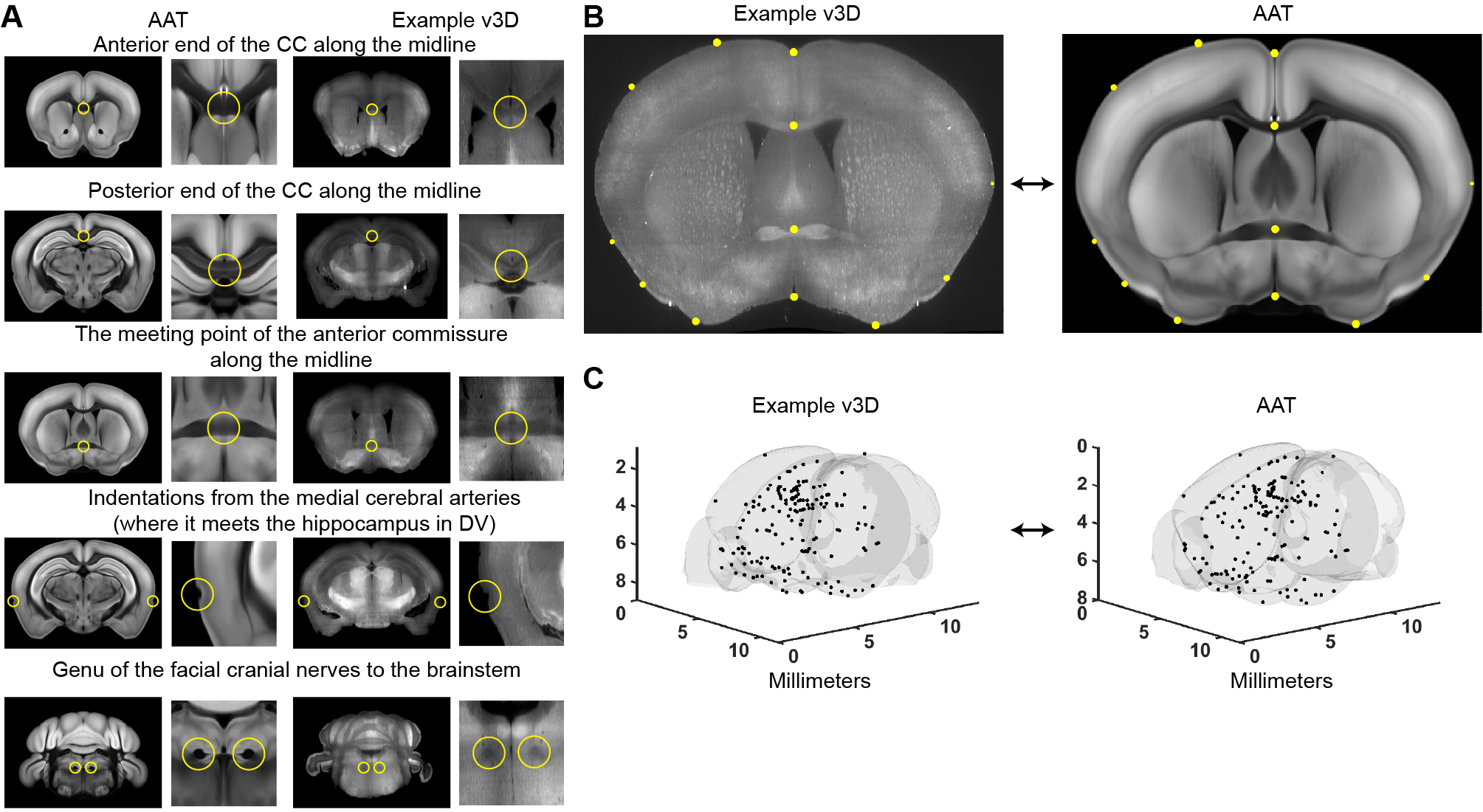

Supplement: Extended Data Figure 4-1 — Example anatomical landmarks. A, Landmarks for alignment (Table 1). The same landmarks (yellow) are identified in the AAT and v3D image volumes. B, After placement of initial landmarks, warping was applied. Additional landmarks (yellow spheres) were then placed to better align the v3D and AAT. Higher densities of landmarks were placed near the probe tracks. C, All landmarks of an example v3D and the AAT (black dots). Download Figure 4-1, TIF file. [file enu-eN-MNT-0241-21-s01.tif]

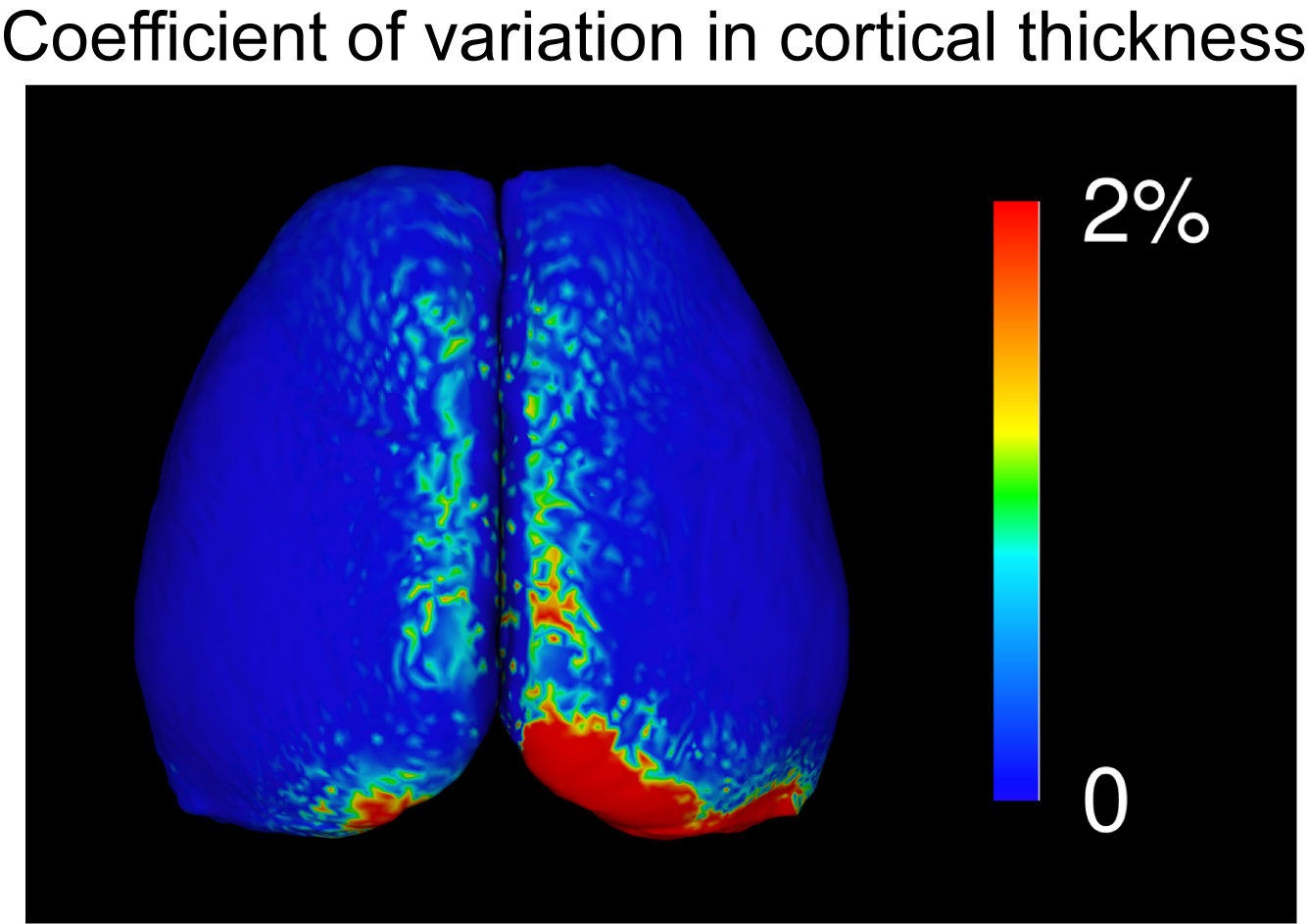

Supplement: Extended Data Figure 4-2 — The variation in the MRI volumes is small across mice. SD as a percentage of the mean thickness measured in the nine VGAT-ChR2-eYFP mice used to generate the MRI3D template (Lerch et al., 2008). Download Figure 4-2, TIF file. [file enu-eN-MNT-0241-21-s02.tif]

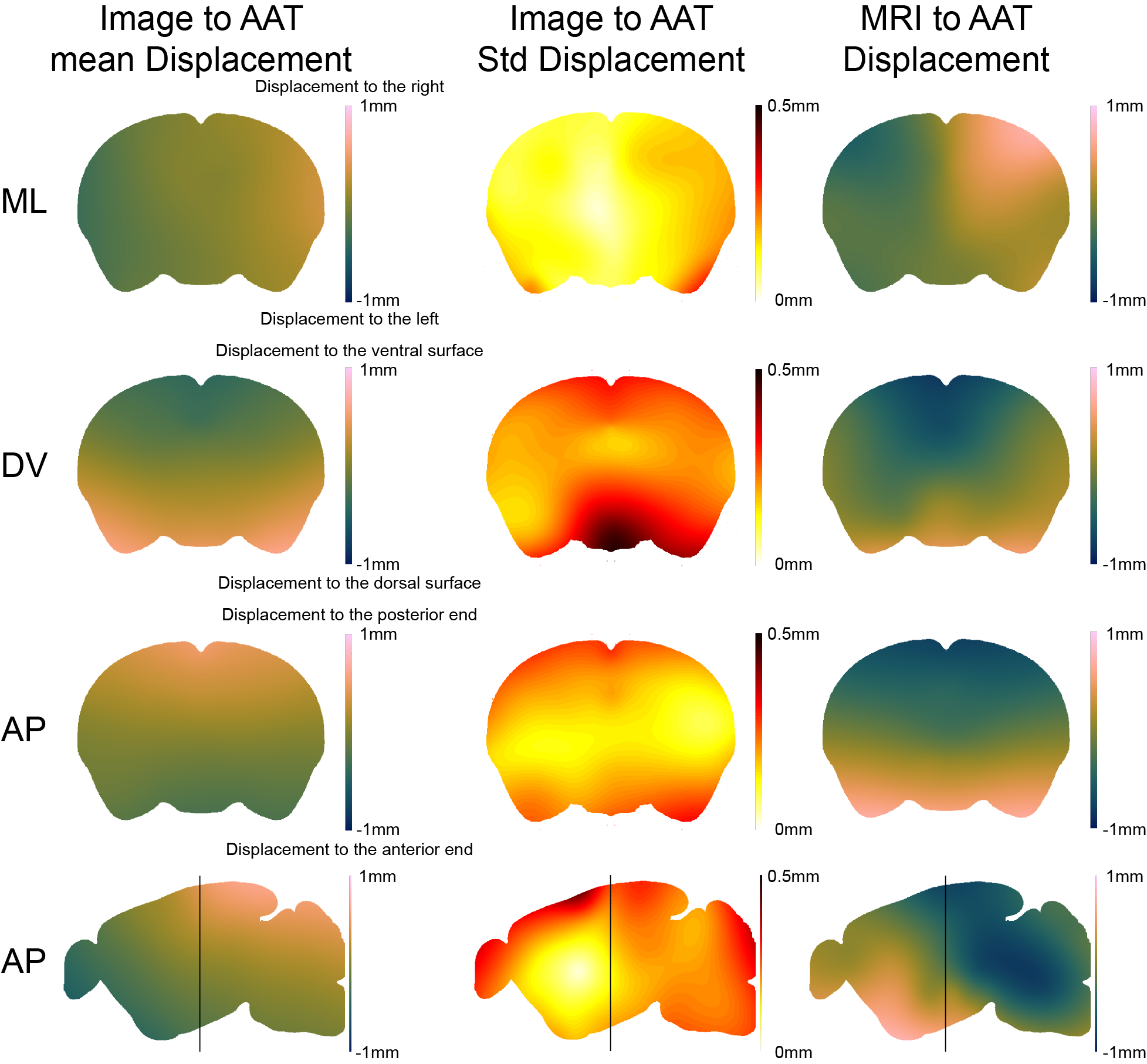

Supplement: Extended Data Figure 4-3 — Example warp fields for one coronal section and one sagittal section. Left, Averaged displacements to warp v3D image volumes onto the AAT. ML, displacements of the v3D along medial-lateral axis; DV, dorsal-ventral; AP, anterior-posterior. For example, in the top left image (ML), the v3D coronal section image has to be stretched laterally to align with the AAT (see Fig. 4A). The black lines on the sagittal sections at the bottom indicate the AP position of the coronal sections. Middle, SD of the displacements required to warp v3D image volumes onto the AAT (9 mice). Right, Displacements to warp the average MRI3D volume onto the AAT. Download Figure 4-3, TIF file. [file enu-eN-MNT-0241-21-s03.tif]

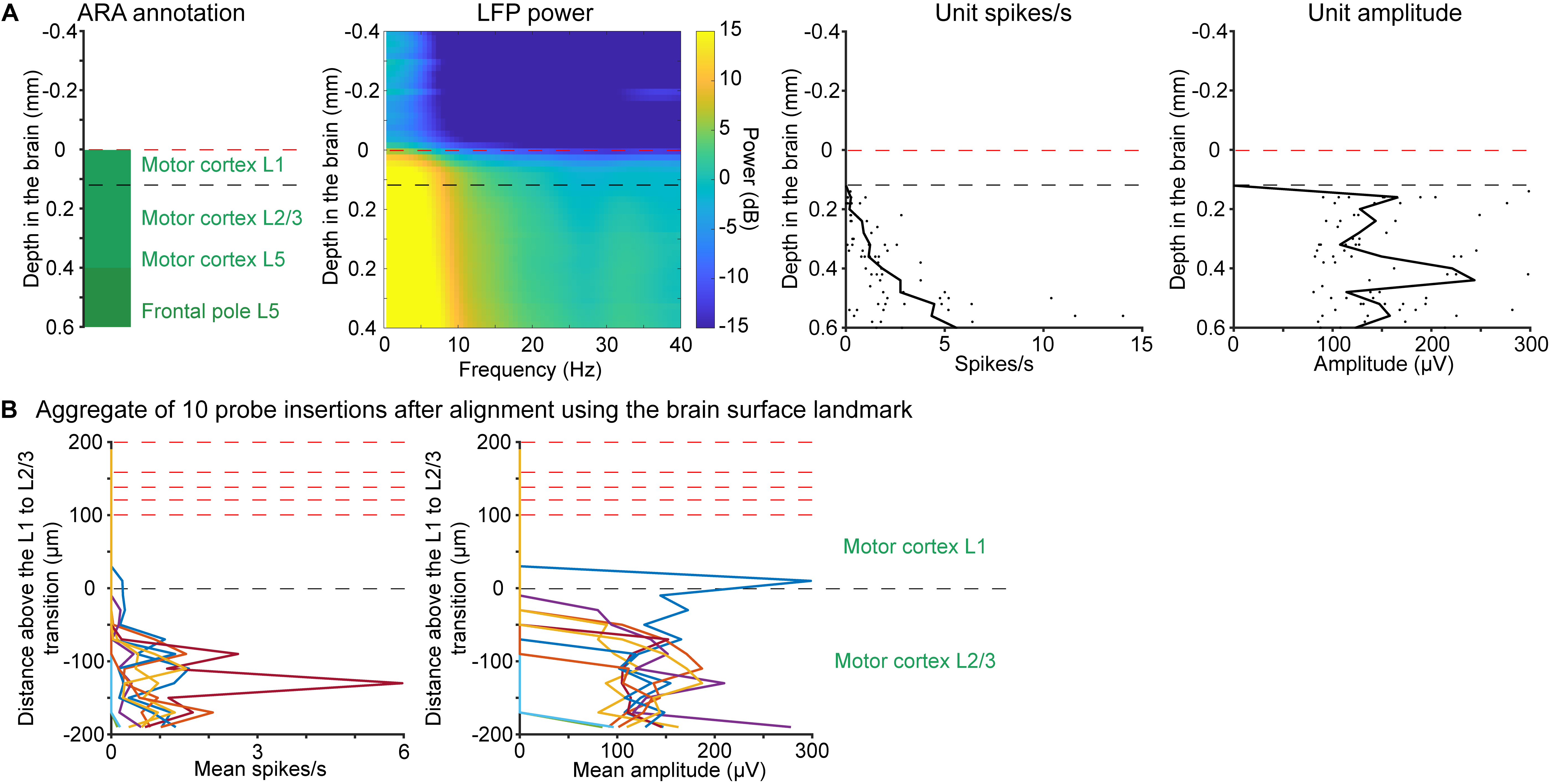

Supplement: Extended Data Figure 7-1 — Cross-validation of the example electrophysiological landmark. A, The ARA annotation along with the LFP power, unit spike rate and unit amplitude across an example probe penetration. The increase in LFP power is an electrophysiological landmark used to localize the electrode at the surface of the brain (red dashed line). The interpolated electrodes beneath the landmark show good agreement at the transition between L1 and L2/3 of the motor cortex (black dashed line), where there is a lack of units at L1. Each dot represents a unit and the line indicates the moving average. B, After aligning the electrodes using the electrophysiological landmark at the surface of the brain. The landmark is cross-validated by aggregating the sites around the transition of L1 to L2/3 for multiple probes. Each color represents the moving average from one probe insertion. Red dash lines indicate the surface of the brain used for alignment. Black dashed line is the transition between L1 and L2/3 of the motor cortex. L1 lacks units above 80 μV. Download Figure 7-1, TIF file. [file enu-eN-MNT-0241-21-s04.tif]

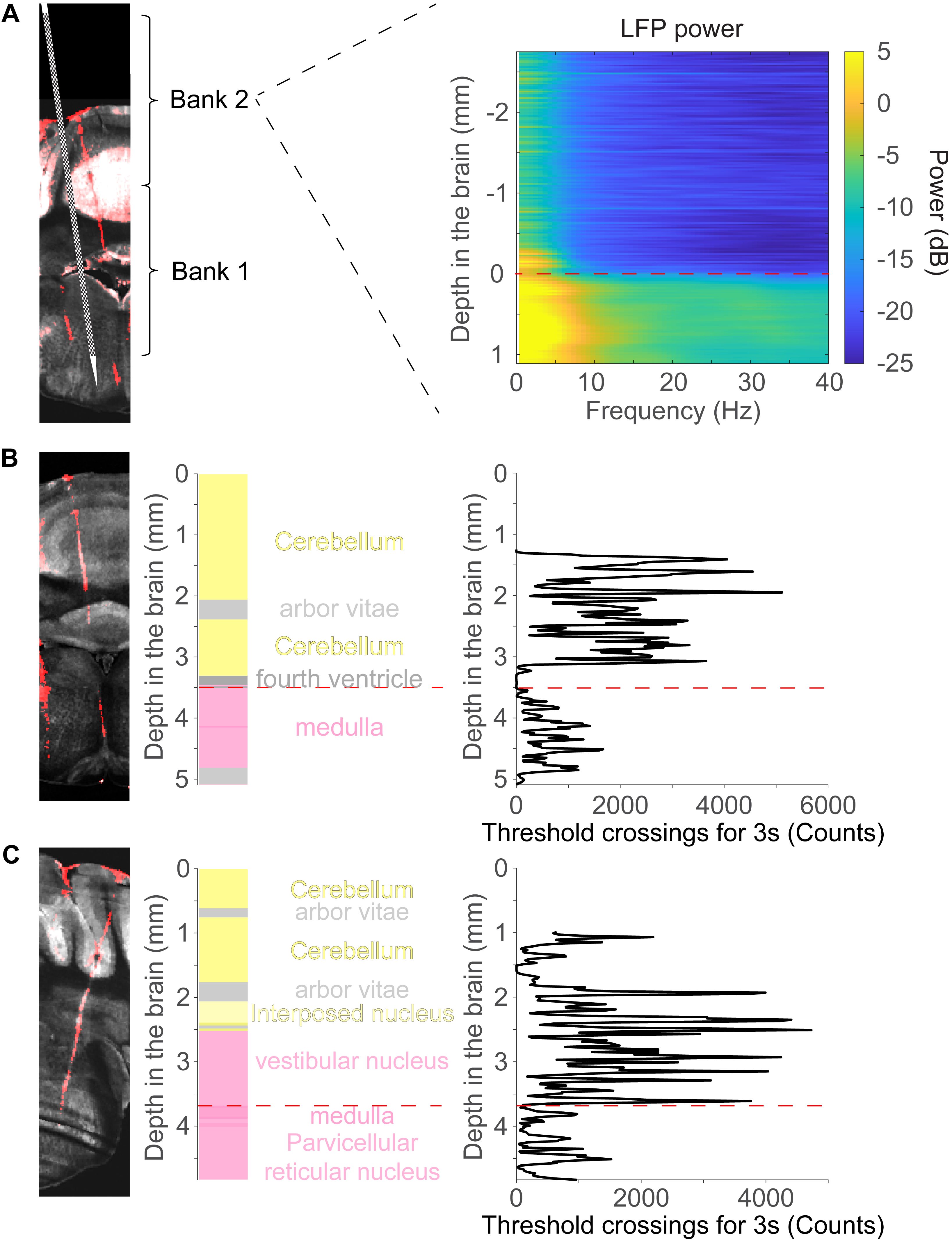

Supplement: Extended Data Figure 7-2 — Additional example electrophysiological landmarks. A, For all penetrations covering a distance longer than the bottom bank of the probe (3.84 mm for Neuropixels 1.0 probes). We can estimate surface by recording from a bank of electrodes above the bottom bank. code_cache/lfpSurface at master · hanhou/code_cache (https://github.com/). B, Example probe insertion passing through the fourth ventricle. C, A probe passing through the vestibular nucleus. The vestibular nucleus in the medulla often has higher firing rate units than other parts of the medulla. Download Figure 7-2, TIF file. [file enu-eN-MNT-0241-21-s05.tif]
